# Rational design of the genetic code expansion toolkit for *in vivo* encoding of D-amino acids

**DOI:** 10.3389/fgene.2023.1277489

**Published:** 2023-10-13

**Authors:** Han-Kai Jiang, Jui-Hung Weng, Yi-Hui Wang, Jo-Chu Tsou, Pei-Jung Chen, An-Li Andrea Ko, Dieter Söll, Ming-Daw Tsai, Yane-Shih Wang

**Affiliations:** ^1^ Institute of Biological Chemistry, Academia Sinica, Taipei, Taiwan; ^2^ Taiwan International Graduate Program Chemical Biology and Molecular Biophysics, Institute of Biological Chemistry, Academia Sinica, Taipei, Taiwan; ^3^ Department of Chemistry, National Tsing Hua University, Hsinchu, Taiwan; ^4^ Institute of Biochemical Sciences, National Taiwan University, Taipei, Taiwan; ^5^ Department of Molecular Biophysics and Biochemistry, Yale University, New Haven, CT, United States; ^6^ Department of Chemistry, Yale University, New Haven, CT, United States

**Keywords:** pyrrolysyl-tRNA synthetase, genetic code expansion, noncanonical amino acids, D-phenylalanine analogs, synthetic biology, amber suppression

## Abstract

Once thought to be non-naturally occurring, D-amino acids (DAAs) have in recent years been revealed to play a wide range of physiological roles across the tree of life, including in human systems. Synthetic biologists have since exploited DAAs’ unique biophysical properties to generate peptides and proteins with novel or enhanced functions. However, while peptides and small proteins containing DAAs can be efficiently prepared *in vitro*, producing large-sized heterochiral proteins poses as a major challenge mainly due to absence of pre-existing DAA translational machinery and presence of endogenous chiral discriminators. Based on our previous work demonstrating pyrrolysyl-tRNA synthetase’s (PylRS’) remarkable substrate polyspecificity, this work attempts to increase PylRS’ ability in directly charging tRNA^Pyl^ with D-phenylalanine analogs (DFAs). We here report a novel, polyspecific *Methanosarcina mazei* PylRS mutant, DFRS2, capable of incorporating DFAs into proteins via ribosomal synthesis *in vivo*. To validate its utility, *in vivo* translational DAA substitution were performed in superfolder green fluorescent protein and human heavy chain ferritin, successfully altering both proteins’ physiochemical properties. Furthermore, aminoacylation kinetic assays further demonstrated aminoacylation of DFAs by DFRS2 *in vitro*.

## 1 Introduction

D-amino acids (DAAs) are widely present in living organisms, in both prokaryotes and eukaryotes (Mothet et al., 2000; Lam et al., 2009). These are naturally encoded by either enzymatic non-ribosomal synthesis or peptide N-terminal post-translational racemization (Kreil, 1997; Lupoli et al., 2011; Huo and van der Donk, 2016), whereas synthetically, DAA-containing peptides can also be efficiently prepared by either the flexible *in vitro* translation (FIT) system or solid-phase peptide synthesis (Fujino et al., 2013; Chang et al., 2015; Levinson et al., 2017). By far, incorporations of DAAs have been reported to endow polypeptides with distinct chirality, allowing them to gain resistance against protease digestion, and providing them with better target binding affinity, thermostability or even the ability to escape from the N-end rule (Tugyi et al., 2005; Welch et al., 2007; Liu et al., 2010; Rodriguez-Granillo et al., 2011; Chen et al., 2013; Rabideau and Pentelute, 2015; Simon et al., 2016; Uppalapati et al., 2016; Gao et al., 2019). However, to this point, protein engineering using DAAs has been largely limited due to endogenous translational machineries’ exclusive preference of L-amino acids (LAAs) over DAAs as building blocks for protein synthesis *in vivo* (Fleisher et al., 2018). *In vitro,* translational apparatuses also appear to have slower incorporation kinetics in charging DAAs over LAAs (Liljeruhm et al., 2019; Melnikov et al., 2019). To overcome these biological constraints, endogenous chiral discriminators (e.g., the ribosome and elongation factor Tu) have been engineered and reconstituted to improve efficiency of DAA incorporation utilizing the FIT system (Dedkova et al., 2003; 2006; Achenbach et al., 2015). In addition, FIT systems equipped with engineered tRNAs and elongation factor Ps have been revealed to foster synthesis of DAA-encoded peptides(Katoh et al., 2017). Nevertheless, DAA incorporation *in vivo* remains challenging. Even though some endogenous aminoacyl-tRNA synthetases (AARSs) may mischarge DAAs, eventually, these would be removed by intrinsic enzymatic racemization or deacylation (Kreil, 1997; Soutourina et al., 2000; Rybak et al., 2019). Hence, to further evolve protein structure and function via non-canonical amino acid (ncAA) incorporations, the development of an efficient DAA-tolerant AARS·tRNA pair to overcome endogenous editing barriers is in need.

**FIGURE 1:**
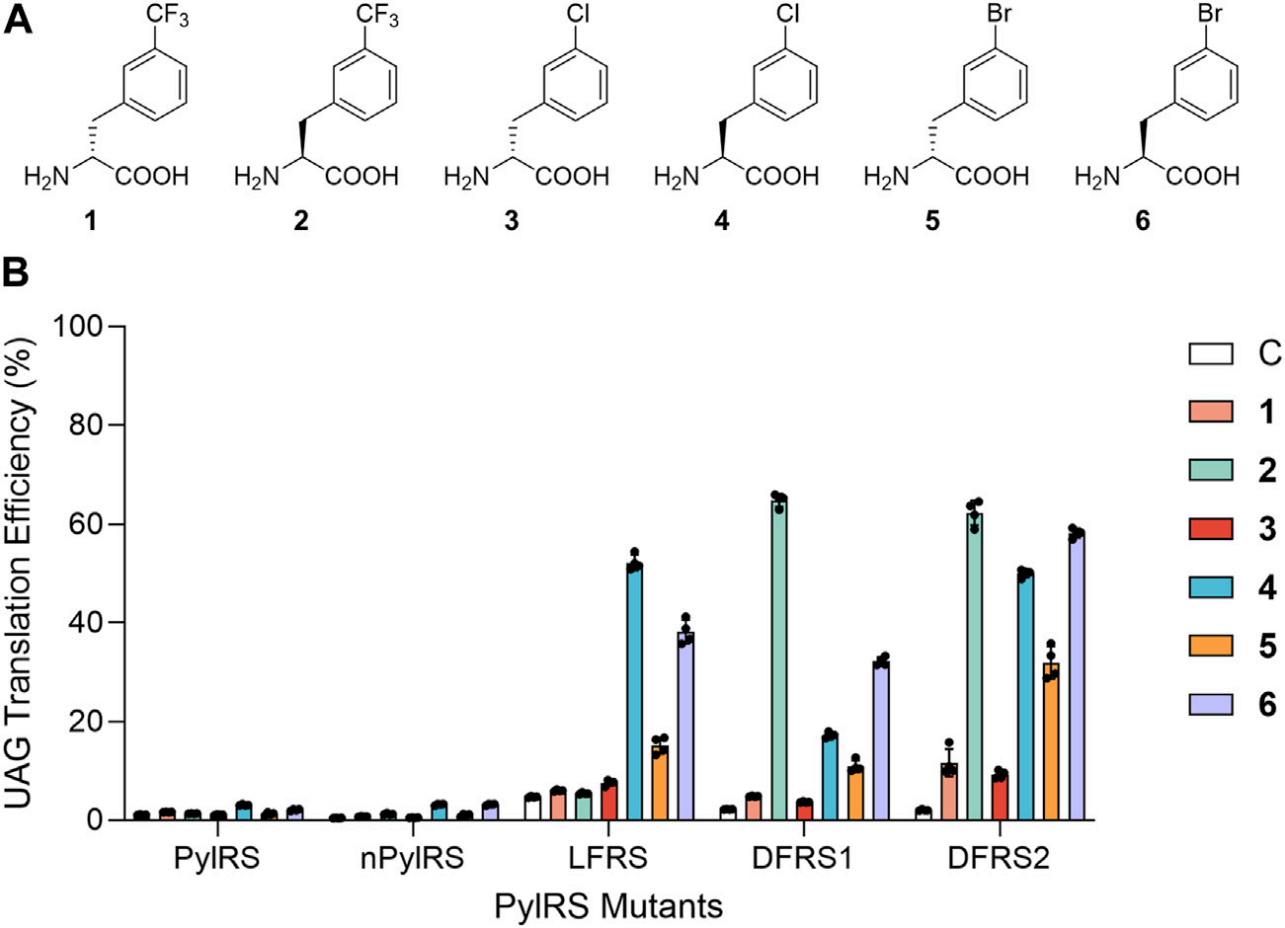
Substrate selectivity of PylRS variants. **(A)** Structures of phenylalanine analogs employed in this study. **(B)** Suppression efficiencies of PylRS variants were examined using an sfGFP-27TAG reporter in BL21 (DE3) cells in GMML medium containing a dedicated ncAA. UAG translation efficiencies were obtained by normalizing fluorescence emission of sfGFP-27TAG according to that of wild-type sfGFP (with the signal of wild-type sfGFP considered to be 100%). Data are presented the mean ± SD for four replicates.

As genetic code expansion continues to develop as a facile tool to generate ncAA-modified proteins with sophisticated functional groups, the catalytic pocket of the pyrrolysyl-tRNA synthetase (PylRS) derived from the archaeal *Methanosarcina mazei* has been extensively engineered (Wang et al., 2016; Yamaguchi et al., 2016; Chen et al., 2019; Wang et al., 2019). Specifically, through amber suppression, evolved PylRSs demonstrate exceptional competence in incorporating various ncAAs into both prokaryotic and eukaryotic proteins (Tsai et al., 2015; Han et al., 2017; Xuan et al., 2017; Yanagisawa et al., 2019; Jang et al., 2020). Entry of DAAs has also been made possible owing to PylRS’ polyspecific active site’s broad substrate scope and diverse ncAA binding mode (Guo et al., 2014). We have previously presented successful results in generating D-lysine analogue-incorporated proteins at low yield using engineered PylRS·tRNA^Pyl^ pairs (Jiang et al., 2020). Interestingly, shown *in vitro*, the centers of ribosome peptidyl transferases are able to maintain 50%–70% relative incorporation efficiencies of D-phenylalanine, D-histidine, and D-tyrosine, in comparison with those of their corresponding L-isomers (Fujino et al., 2013). We therefore hypothesize that by generating excess amounts of D-aminoacyl-tRNA *in vivo*, the cellular editing capacity could be overwhelmed in its role in removing incorporated DAAs. With this in mind, and based on known mutations (i.e., N346A and C348A) that could allow PylRSs to charge tRNA^Pyl^ with L-phenylalanine analogs (LFAs) (Wang et al., 2012; Wang et al., 2013), here, we expand the polyspecificity of the PylRS·tRNA^Pyl^ system via rational design in charging three selected L-phenylalanine analogs (LFAs) and their corresponding D-phenylalanine analogs (DFAs). That is, D- and L-3-trifluoromethylphenylalanine (**1** and **2**); D- and L-3-chlorophenylalanine (**3** and **4**); and D- and L-3-bromophenylalanine (**5** and **6**) (Figure 1A; Supplementary Table S1).

## 2 Materials and methods

### 2.1 General strains and *in vivo* UAG readthrough assay

The ncAAs **1**–**6** were purchased from Chem-Impex Inc. (Wood Dale, IL, United States). Polymerase chain reactions (PCRs) were performed using the KOD Hot Start Polymerase kit (Merck, Bedford, MA, United States). The oligonucleotide synthesis and DNA sequencing services were provided by Genomics Inc. (Taipei, Taiwan). The gene and protein sequences, plasmid constructions of PylRS variants (nPylRS, LFRS, DFRS1, DFRS2, and DFRSc), sfGFP variants, and FTH1 variants, and their respective primers are described in the Supplementary Material.

To monitor stop codon readthrough, pET plasmids containing an sfGFP-27TAG and a tRNA^Pyl^ were co-transformed with a pCDF plasmids containing an *M. mazei* PylRS mutant into *E. coli* BL21 (DE3) competent cells. Cells were recovered in 1 mL LB medium at 37°C for 1 h and plated on a LB agar plate containing Amp (100 μg/mL) and Sp (100 μg/mL). A single colony was selected from the plate and cultured in 1 mL LB medium at 37°C overnight. The cultured bacteria were then transferred to 50 mL fresh LB medium and incubated at 37°C until OD_595_ reached 0.6–0.8. Cells were harvested, washed twice with GMML medium (M9 salt solution, 1% glycerol, 2 mM MgSO_4_ and 0.1 mM CaCl_2_), and resuspended in GMML medium containing 1 mM IPTG. Aliquots (50 μL) of cell suspension were loaded into a 384-well plate (Corning) supplemented with 1 mM of one of the six ncAAs (**1**–**6**). Cells were incubated in a plate reader (BioTek) at 37°C, and the fluorescence intensity (i.e., excitation at 485 nm and emission at 535 nm) as well as OD_595_ were continuously measured for 12 h. The fluorescence intensity of sfGFP was divided by OD_595_ at the 12 h time point, followed by subtraction of the emission signal at the 0 h time point to generate relative fluorescence intensity. Normalization of UAG translation efficiency was calculated using the relative fluorescence intensity of sfGFP-27TAG and wild-type sfGFP (Ko et al., 2013; Fan et al., 2014; Jiang et al., 2020). The experiments were performed with four biological replicates.

### 2.2 Expression and purification of sfGFP and FTH1 variants

To express ncAA incorporated superfolder green fluorescent protein (sfGFP) or human heavy chain ferritin (FTH1), a pET plasmids containing sfGFP-XTAG (X denotes a TAG codon at either position 27 or 66), FTH1-1xTAG (x denotes a TAG codon at position 60) or FTH1-2xTAG (x denotes two TAG codons at positions 60 and 67), and *M. mazei* tRNA^Pyl^ were co-transformed with a pCDF plasmids harboring an *M. mazei* PylRS mutant into *Escherichia coli* BL21 (DE3) competent cells. After 1 h recovery at 37°C, cells were spread on LB agar plates containing ampicillin (Amp, 100 μg/mL) and streptomycin (Sp, 100 μg/mL). Single colonies were selected and grown overnight in 5 mL cultures. 1 mL of cultured bacteria was then transferred to 100 mL of LB medium, growing at 37 °C until OD_595_ reached 0.6–0.8. Cells were centrifuged at 6,000 x g for 10 min and the supernatant was discarded. Pellets were washed by GMML medium twice and proteins were expressed in 100 mL of GMML medium (M9 salt solution, 1% glycerol, 2 mM MgSO_4_, and 0.1 mM CaCl_2_) containing 1 mM IPTG and an ncAA at 37°C for 12 h. Cells were centrifuged at 6,000 × g for 30 min at 4°C, resuspended in lysis buffer (1X Phosphate-Buffered Saline (PBS), pH 7.4), and lysed by sonication. The cell lysate was centrifuged at 20,000 × g for 45 min at 4°C and the supernatant was transferred to an open column containing 0.5 mL Ni-NTA resin (Roche). The resin was washed with 10 column volumes of lysis buffer (1X PBS, pH 7.4) and 5 column volumes of wash buffer (1X PBS, 5 mM imidazole, pH 7.4). Finally, target proteins were eluted with 5 column volumes of elution buffer (1X PBS, 200 mM imidazole, pH 7.4). The buffer was changed using Amicon Ultra-15 Centrifugal Filters (MWCO 10 kDa) by concentrating the protein samples, then reconstituting the concentrate in PBS buffer. Purified proteins were analyzed by 12% sodium dodecyl sulfate polyacrylamide gel electrophoresis (SDS-PAGE), distributed to Eppendorf and stored at −80°C.

### 2.3 Expression and purification of DFRSc for crystallization

To express DFRSc, a pET plasmid containing DFRS2^185−454^(DFRSc) was transformed into *E. coli* BL21 (DE3) competent cells. Cells were recovered in 1 mL LB medium at 37°C for 1 h and plated on a LB agar plate containing Amp (100 μg/mL). A single colony was chosen and cultured in 10 mL LB medium at 37°C overnight. The cultured bacteria were transferred to 1 L sterilize LB medium and incubated at 37°C until OD_595_ reached 0.6–0.8. The protein was induced with 0.5 mM IPTG at 25°C overnight. Cells were collected, resuspended in buffer A (50 mM KH_2_PO_4_/K_2_HPO_4_, 500 mM NaCl, 25 mM imidazole, 5 mM β-ME, 10% glycerol, and 0.1 mM PMSF at pH 7.4), and lysed by sonication. The cell lysate was clarified by centrifugation at 20,000 × g for 1 h at 4°C. The supernatant was collected, subjected to an open column equipped with 10 mL Ni-NTA resin (Roche), and washed by 10 column volumes of buffer A. The protein was eluted by buffer A containing 300 mM imidazole. The sample was concentrated using Amicon Ultra-15 Centrifugal Filters (MWCO 10 kDa) and subjected to a size-exclusion chromatography (SEC) HiLoad 16/600 Superdex 75 pg column (GE Healthcare) equilibrated with buffer B (25 mM KH_2_PO_4_/K_2_HPO_4_, 1 mM DTT, 10% glycerol, and 0.1 mM PMSF, pH 7.4). The eluted fractions were then subjected to an anion exchange Mono Q column (GE Healthcare) with a 0%–80% (v/v) gradient of 1 M NaCl. The protein fractions were concentrated using Amicon Ultra-15 Centrifugal Filters (MWCO 10 kDa) and, again, loaded into the SEC column, eluted using buffer C (20 mM KH_2_PO_4_/K_2_HPO_4_, 300 mM NaCl, 5 mM MgCl_2_, and 10 mM β-ME, pH 7.4) for crystallization. The DFRSc fractions were collected and concentrated using Amicon Ultra-15 Centrifugal Filters (MWCO 10 kDa). The purified protein was analyzed by SDS-PAGE, distributed to Eppendorf and stored at −80°C (Yanagisawa et al., 2006).

### 2.4 Protein electrospray ionization mass spectrometry (ESI-MS) analysis

The pure protein was diluted with 50% acetonitrile and 1% formic acid. An aliquot corresponding to 1 ρmol of the pure protein was injected via an ESI source (Waters LockSpray Exact Mass Ionization Source) with a syringe pump (Harvard Apparatus, MA), holding a flow rate of 5 μL/min throughout the analysis. The mass of intact proteins was determined using the Waters Synapt G2 HDMS mass spectrometer (Waters, Milford, MA). The acquired spectra were deconvoluted to single-charge states using the MaxEnt1 algorithm of the MassLynx 4.1 software (Waters).

### 2.5 X-crystal structural analysis

Crystals of DFRSc (12 mg/mL) with 5 mM AMP-PNP were obtained at 293 K by the hanging drop vapor-diffusion method in 100 mM HEPES buffer at pH 7.5, with 10% PEG 8000% and 10% ethylene glycerol. For soaking, 5 mM ncAA was added 1 h before collecting the crystals. 20% glycerol was added to the crystallization buffer as a cryoprotectant before removing the crystals from crystallization droplets. Molecular replacements were carried out using a previously reported PylRS (PDB: 2ZCE) structure as a search model through PHENIX-Phaser-MR (Adams et al., 2010). Final models were built after several runs of refinement in phenix.refine (Langer et al., 2008) and manual re-building in Coot (Emsley et al., 2010). The final models were analyzed with PROCHECK (Laskowski et al., 1993). Structural presentations were generated using the PyMOL Molecular Graphics System (Version 1.7.4 Schrödinger, LLC).

### 2.6 Circular dichroism spectroscopy

Wild-type sfGFP and sfGFP-Y66-**3**, **4** (0.4 mg/mL) were prepared in 5 mM sodium phosphate buffer (pH 7.4). CD spectra were collected by the J-815 Circular Dichroism Spectropolarimeter (Jasco). Experiments were performed using a 1 mm path-length cuvette. Denaturation midpoint experiments were carried out by monitoring the change in CD spectra from 195 to 260 nm with temperature increase from 25°C to 95°C with either a 10 or 2°C interval. The melting curves were analyzed in transition at 205 nm with a 1°C interval. Thermal Denaturation Multi Analysis [on thermodynamics parameters including the denaturing temperature (Tm), enthalpy (ΔH) and entropy (ΔS)] was done using the Jasco software.

### 2.7 Dynamic light scattering analysis

FTH1 samples were prepared in 1X PBS to final concertation of 0.3–0.5 mg/mL before filtering to remove aggregation or dust in solution. Experiments were conducted using the nanoparticle analyzer (Malvern Zetasizer Nano ZS) with 1 mL disposable cuvettes at 25°C in triplicate.

### 2.8 Transmission electron microscopy (TEM) analysis

FTH1 samples were prepared in 1X PBS (pH 8.0) to final concentrations of 0.075 mg/mL 5 μL of samples were dropped onto TEM grids (formvar/carbon 400 mesh, copper) followed by glow discharge treatments (Emitech K100X, 25 mA for 30 s). After air drying for 90 s, excess solution was wicked with filter paper. Next, the grids were rinsed with 5 µL of distilled deionized water followed by wicking with filter paper. This step was repeated in triplicate. 5 μL filtered 1% uranyl acetate was dropped onto grids, incubated for 60 s, then wicked with filter paper. The grids were kept in a grid-box and stored in an electronic dry cabinet until the grids were fully dried. The TEM images of FTH1 variants were taken by the FEI Tecnai G2 TF20 Super-Twin. The accelerating voltage was set as 120 kV.

### 2.9 Absorption and emission spectra analysis

sfGFP-Y66 variants were diluted to final concentrations of 0.5 mg/mL in PBS at pH 7.4. Spectra were collected using Duetta (Horiba) with 3 mm quartz cuvettes at 25°C. The scanning wavelength of absorption spectra started from 250 to 700 nm with 5 nm intervals. The emission spectra of sfGFP-Y66 and wild-type variants were recorded from 380 to 750 and from 500 to 750 nm, respectively, with 5 nm intervals.

### 2.10 Preparation of radiolabeled tRNA^Pyl^

*In vitro* tRNA^Pyl^ transcription reactions were carried out using purified T7 RNA polymerase and synthetic oligonucleotides following the previously reported protocol (Ellinger and Ehricht, 1998; Korencic et al., 2002). The oligonucleotides were synthesized by the W. M. Keck Biotechnology Resource Laboratory at Yale University. Transcription reactions were carried out with reaction mixtures of 40 μg DNA template, 10 mM Tris·HCl (pH 8), 20 mM MgCl_2_, 2 μg/mL pyrophosphatase, 1 mM spermidine, 0.01% Triton X-100, 5 μg/mL bovine serum albumin (BSA), 5 mM DTT, 4 mM NTP mix along with T7 RNA polymerase at 37°C for 6 h tRNA transcripts were purified using 12% urea-polyacrylamide gels and extracted with 500 mM ammonium acetate buffer (pH 5.2). [α-^32^P]-ATP (PerkinElmer) was used in the reaction to label tRNA^Pyl^ transcripts at the 3′-end adenosines using the *E. coli* CCA-adding enzyme as previously described (Ledoux and Uhlenbeck, 2008).

### 2.11 ATP-[^32^P] PP_i_ exchange assay

DFRSc was used in the reaction to measure the kinetics of amino acid activation. Reactions (final volumes of 20 µL) were performed at 37°C in 150 mM Na-HEPES (pH 7.2), 10 mM kF, 10 mM MgCl_2_, 50 mM KCl, 0.2 mg/mL BSA, 10 mM DTT, 2 mM ATP, 1 mM PP_i_, 0.2 µCi [γ-^32^P]-ATP, 10 µM DFRSc and ncAAs **1**-**6** in different concentration ranges. Reactions were collected (1 µL for each) over time (0, 1, 2, 3, 4, 5 min) and spotted onto TLC PEI cellulose F plates (Merck). TLC plates were developed in a running buffer (1 M KH_2_PO_4_ and 4 M urea, pH 3.5) for approximately 25 min to separate ATP and PP_i_. The air-dried plates were exposed on image plates, scanned on Amersham Typhoon Biomolecular Imager, and quantified using ImageJ software (Guo et al., 2014).

### 2.12 Aminoacylation of tRNA^Pyl^

*In vitro* aminoacylation assays were modified from previously described protocols (Guo et al., 2014; Guo et al., 2022). Aminoacylation reactions (final volumes of 15 μL) were carried out at 37°C in 100 mM Na-HEPES (pH 7.2), 25 mM MgCl_2_, 60 mM NaCl, 5 mM ATP, 1 mM DTT, 1 µM full-length DFRS2, 15 µM tRNA (with trace amounts of [α-^32^P]-labeled tRNA^Pyl^), and ncAAs **1**-**6** in different concentration ranges. Reactions were collected (2 µL for each) over time (LFAs **2**, **4** and **6**: 0, 2, 4, 6, 8, 10 min; DFAs **1**, **3** and **5**: 0, 5, 10, 15, 20, 25 min) and quenched by adding 5 μL of buffer containing 200 mM sodium acetate (pH 5.2) with 0.1 U/μL of nuclease P1 (Millipore-Sigma). The reaction mixtures were incubated at room temperature for 30 min 2 μL of reaction mixtures were spotted onto TLC PEI cellulose F plates, and the TLC plates were developed in a running buffer (0.1 M sodium acetate and 5% acetic acid) for approximately 25 min to separate [α-^32^P] AMP and aminoacyl-[α-^32^P] AMP. The air-dried plates were exposed on image plates, scanned on the Amersham Typhoon Biomolecular Imager, and quantified using the ImageJ software.

## 3 Results

### 3.1 Rational design of PylRS in recognizing D- and LFAs

To examine the ability of PylRS’ active site in recognizing D- and LFAs, we measured UAG suppression efficiency in *E. coli* cells that were simultaneously expressing either the wild-type PylRS·tRNA^Pyl^ or the nPylRS·tRNA^Pyl^ pair, an sfGFP that contains an internal UAG codon at position 27 (sfGFP-27TAG), and one of the aforementioned six ncAAs (Figure 1A). The chiral purities of these ncAAs were confirmed via HPLC (Supplementary Table S1–S3). nPylRS is an efficient PylRS variant which contains three extra mutations on its tRNA-binding domain (Supplementary Table S1) (Jiang et al., 2020). For both PylRS and nPylRS, we observed notable increases in sfGFP expression when LFAs **4** and **6** were added to cell cultures, suggesting their intact active sites to be capable of charging LFAs despite the low UAG readthrough efficiency. As previous works suggested, while the N346A/C348A PylRS mutant is highly active in charging a number of LFAs, it exhibits much less activity in incorporating L-phenylalanine. It is thought that the amide group of N346 in wild-type PylRS plays an important role in excluding L-phenylalanine from the active site (Wang et al., 2013). To forge a pocket that fits both D- and LFAs, we generated a PylRS mutant containing a single N346 V mutation, namely, LFRS, so to study if increasing the hydrophobicity of N346 would enhance its binding affinity to hydrophobic phenylalanine analogs (Supplementary Table S1).

By analyzing the emitted fluorescence of the sfGFP-27TAG reporter relative to that of wild-type sfGFP, the UAG codon readthrough rates in the presence of LFAs **4** and **6** by LFRS were determined to be 52.2% and 38.1%, respectively (Figure 1B). In addition, UAG suppression rate by LFRS in the presence of DFA **5** was 15.1%, demonstrating the significance of the N346 V mutation in binding of not only LFAs but also DFAs to PylRS’ amino acid binding site. Accordingly, since the active site of another PylRS mutant, BtaRS, is known to be capable of recognizing diverse *meta*-substituted LFAs (Englert et al., 2015), BtaRS’ promiscuous pocket that harbors the N346G and C348Q mutations may also present it as a good candidate for further engineering to accommodate DFAs. On the other hand, the hydrophobic side chain of residue V401 was revealed to preclude L-tryptophan from entering BtaRS (Englert et al., 2015). To expand the polyspecificity of PylRS, we sought to mutate V401 to glycine at BtaRS’ active site, generating a PylRS N346G/C348Q/V401G mutant, DFRS1 (Supplementary Table S1). Moreover, by transplanting the R61K, H63Y and S193R mutations found to improve PylRS activity to DFRS1 (Jiang et al., 2020), we generated a second variant, DFRS2. We sought to compare the limited L-phenylalanine (LPhe) affinity of PylRS-N346A/C348A mutant. The four PylRS mutants in the study were then tested against nineteen LAAs and DAAs in amber stop codon suppression assay (Supplementary Figure S4). In the emitted fluorescence signals of sfGFP-27TAG reporter production, N-PylRS, and DFRS1 show no activity against LAAs or DAAs. The other PylRS mutants, LFRS and DFRS2, show limited activity in charging LPhe as a similar property of PylRS-N346A/C348A mutant. Obviously, additional R61K, H63Y and S193R mutations of DFRS2 contribute to the limited activity in charging LPhe compared with DFRS1. Again, by referencing to the emitted fluorescence of the sfGFP-27TAG reporter and comparing it with that of wild-type sfGFP, DFRS1 displayed UAG readthrough rates of 64.8%, 17.2% and 32.2% in the presence of LFAs **2**, **4**, and **6**, respectively (Figure 1B). By contrast, DFRS1 showed a UAG suppression rate of only 10.9% with DFA **5**. As for DFRS2, we observed a significant increase in fluorescence in the presence of LFAs **2**, **4**, and **6**, revealing amber stop codon suppression rates of 62.3%, 50.0% and 58.2%, respectively. Furthermore, DFRS2 yielded noteworthy UAG translation rates of DFAs **1**, **3**, and **5**, being 11.6%, 9.2% and 31.9%, respectively.

### 3.2 Measuring *in vitro* kinetics of DFRS2

Similar to other AARSs, PylRS catalyzes a two-step reaction to load amino acids onto its cognate tRNA^Pyl^s (Guo et al., 2014). ATP-PP_i_ exchange assays allow monitoring of the first step, being the adenylation of amino acids, whereas aminoacylation assays examine the second, being the formation of aminoacylated tRNA products through radiolabeling (Wolfson et al., 1998; Polycarpo et al., 2006; Guo et al., 2014). To better understand whether the chiral phenylalanine analogs are biochemically acylated onto suppressing tRNA^Pyl^ by DFRS2·tRNA^Pyl^ pairs, we examined the aminoacylation activity of DFRS2 with D- and LFAs *in vitro* through tRNA^Pyl^’s ligation efficiency. Seeing that the poorly soluble PylRS N-terminal domain is not essential for amino acid activation (Herring et al., 2007), we employed a truncated form of DFRS2, DFRSc (residues 185-454), in our ATP-PP_i_ exchange assays (Table 1). As a positive control, we first determined the Michaelis-Menten constant (*K*_*M*_) and catalytic constant (*k*_cat_) of the truncated PylRS (residues 185-454), PylRSc, to BocK, being 0.84 ± 0.06 mM and 12.93 ± 0.62 s^-1^ × 10^−2^, respectively. Purified DFRSc were incubated with varying concentrations of ncAAs **1**-**6** in the presence of radiolabeled ATP. Our results revealed the *K*_*M*_ of DFRSc to LFA **4** (5.38 ± 1.28 mM) to be lower than those to LFAs **2** (7.12 ± 1.67 mM) and **6** (9.80 ± 1.65 mM), whereas the *k*_cat_ of DFRSc with LFAs **2**, **4**, and **6** (10.43 ± 0.37, 11.18 ± 0.29, and 10.21 ± 0.35 s^-1^ × 10^−2^, respectively) were similar. Unfortunately, due to DFAs’ low solubility limits, the *K*_*M*_ and *k*_cat_ of DFRSc to all three DFAs were undetectable. The relative catalytic efficiencies (*k*_cat_/*K*_*M*_) of DFRSc with LFAs were then evaluated in reference to that of PylRSc to BocK, revealing DFRSc’s amino acid activation efficiency to be 15 to 32 folds lower than that of PylRSc.

**TABLE 1:** Apparent kinetic parameters of PylRS variants for amino acid activation.

Enzymes	Amino acids	*K*_*M*_, μM × 10^3^	*k*_cat_, s^-1^ × 10^−2^	*k*_cat_ */K*_*M*_, μM^-1^∙s^-1^ × 10^−5^	Relative catalytic efficiency
PylRSc	BocK	0.84 ± 0.06	12.93 ± 0.62	15.39	100
DFRSc	D-CF_3_-Phe (**1**)	ND	ND	—	—
	L-CF_3_-Phe (**2**)	7.12 ± 1.67	10.43 ± 0.37	1.46	9.5
	D-Cl-Phe (**3**)	ND	ND	—	—
	L-Cl-Phe (**4**)	5.38 ± 1.28	11.18 ± 0.29	2.08	13.5
	D-Br-Phe (**5**)	ND	ND	—	—
	L-Br-Phe (**6**)	9.80 ± 1.65	10.21 ± 0.35	1.04	6.8

ND, not detectable.

Data are shown as the mean ± SD, of three technical replicates.

In parallel, aminoacylation assays were carried out *in vitro* with varying concentrations of ncAAs **1**-**6** in the presence of radiolabeled tRNA^Pyl^ and full-length DFRS2, so to determine the enzyme’s full ligase activity (Table 2) (Guo et al., 2014). Results indicated DFRS2 to display prominent aminoacylation activity with LFAs **2**, **4**, and **6**, exhibiting *K*_*M*_ of 0.09 ± 0.012, 0.05 ± 0.004, and 0.12 ± 0.010 mM, respectively. By contrast, DFRS2 showed notably higher *K*_*M*_ with DFAs **1**, **3** and **5** (1.65 ± 0.210, 1.20 ± 0.112 and 0.81 ± 0.164 mM, respectively). Moreover, DFRS2 exhibited lower *k*_cat_ with DFAs **1**, **3**, and **5** (0.07 ± 0.001, 0.11 ± 0.005, and 0.10 ± 0.006 s^-1^ × 10^−2^, respectively) than with LFAs **2**, **4**, and **6** (0.49 ± 0.035, 0.23 ± 0.005, and 0.33 ± 0.015 s^-1^ × 10^−2^, respectively). Again, as a positive control, kinetics measurements of full-length PylRS with BocK were also taken, revealing a *K*_*M*_ of 0.46 ± 0.084 mM and a *k*_cat_ of 1.10 ± 0.043 s^-1^ × 10^−2^. The relative aminoacylation efficiencies of DFRS2 with the ncAAs were then calculated based on the *k*_cat_/*K*_*M*_ of PylRS with BocK, indicating the catalytic efficiency of DFRS2 with LFAs to be 1.2 to 2.3 folds higher, and contrastingly, those with DFAs to be 20 to 59 folds lower than PylRS with BocK.

**TABLE 2:** Apparent kinetic parameters of PylRS variants for aminoacylation.

Enzymes	Amino acids	*K*_*M*_, μM × 10^3^	*k*_cat_, s^-1^ × 10^−2^	*k*_cat_ */K*_*M*_, μM^-1^∙s^-1^ × 10^−5^	Relative catalytic efficiency
PylRS	BocK	0.46 ± 0.084	1.10 ± 0.043	2.39	100
DFRS2	D-CF_3_-Phe (**1**)	1.65 ± 0.210	0.07 ± 0.001	0.04	1.7
	L-CF_3_-Phe (**2**)	0.09 ± 0.012	0.49 ± 0.035	5.44	228
	D-Cl-Phe (**3**)	1.20 ± 0.112	0.11 ± 0.005	0.09	3.8
	L-Cl-Phe (**4**)	0.05 ± 0.004	0.23 ± 0.005	4.60	192
	D-Br-Phe (**5**)	0.81 ± 0.164	0.10 ± 0.006	0.12	5.0
	L-Br-Phe (**6**)	0.12 ± 0.010	0.33 ± 0.015	2.75	115

Data are shown as the mean ± SD, of three technical replicates.

### 3.3 Structural analysis of D- and LFA-bound DFRSc complexes

**FIGURE 2:**
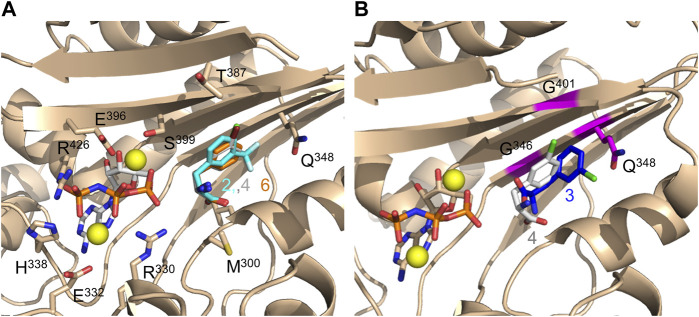
Crystal structures of DFRSc complexed with AMP-PNP and LFA/DFA. **(A)** Three overlapping crystal structures of LFA (**2**, blue stick; **4**, white stick; **6**, orange stick)-bound DFRSc. **(B)** The overlapping crystal structures of DFRSc complexed with D-3-chlorophenylalanine (**3**, blue stick) and L-3-chlorophenylalanine (**4**, white stick). Mutations N346G, C348Q and V401G are presented in magenta. The resolution of each crystal: **2**: 2.2 Å; **3**: 1.9 Å; **4**: 1.9 Å; **6**: 2.5 Å.

We continued to determine the crystal structures of the DFRSc in complex with adenylyl-imidodiphosphate (AMP-PNP) and each of the six ncAAs **1**-**6** at 1.8–2.5 Å resolution (Supplementary Figure S5; Supplementary Table S2). These structures highly resemble the previously reported structures of PylRS’ catalytic domain (Kavran et al., 2007; Yanagisawa et al., 2008). Clear and well-defined electron density maps were also obtained from complexes between LFAs (**2**, **4**, and **6**) and DFRSc, exhibiting similar binding modes (Figure 2A; Supplementary Figure S5D–F). By contrast, only blurred electron density maps were obtained from DFAs (**1**, **3**, or **5**)·DFRSc complexes (Supplementary Figure S5A–C), showing that the binding of these DFAs are less stable than those of their corresponding L-enantiomers (**2**, **4**, and **6**). We observed in the structures of LFA (**2**, **4**, or **6**)-bound DFRSc complexes that the carboxyl groups of LFAs form hydrogen bonds with residues L310 and A302 of DFRSc (Supplementary Figure S5D–F). These hydrogen bonds, however, were disrupted in the structures of DFA (**1**, **3**, or **5**)-bound DFRSc complexes (Supplementary Figure S5A–C). The distance between the carboxyl group of DFA **1** and residues L301 and A302 of DFRSc are 5.3 and 5.7 Å, respectively. In addition, the carboxyl group of DFA **1** forms a hydrogen bond with S399 of DFRSc. In DFA **3** and **5**·DFRSc complexes, however, the amino and carboxyl groups of these DFA main chains were oriented differently from that of the LFAs·DFRSc complexes. Moreover, the superimposition of DFA **3** and LFA **4**·DFRSc structures reveal different binding modes. Particularly, the side chain of DFA **3** was orientated perpendicularly at 95° in relation to that of LFA **4** (Figure 2B).

### 3.4 Biophysical analysis of D- and LFA-encoded sfGFP fluorophores

Maturation of the sfGFP chromophore involves cyclization of the tripeptide T65-Y66-G67, which is responsible for its emission of fluorescence (Pédelacq et al., 2006). Mutations at Y66, which serves as the chromophore’s vital electron donor, are known to affect the spectral properties of sfGFP. Therefore, we examined the spectral characteristics of sfGFP variants where D/LFAs were introduced via the DFRS2·tRNA^Pyl^ pair. The incorporations of ncAAs **1**-**6** into sfGFP containing an internal UAG codon at position 66 (sfGFP-Y66TAG) were validated by ESI-MS analyses (Supplementary Figure S6). sfGFP-Y66-**1**-**6** exhibited blue-shifted excitation and emission fluorescence.

**FIGURE 3:**
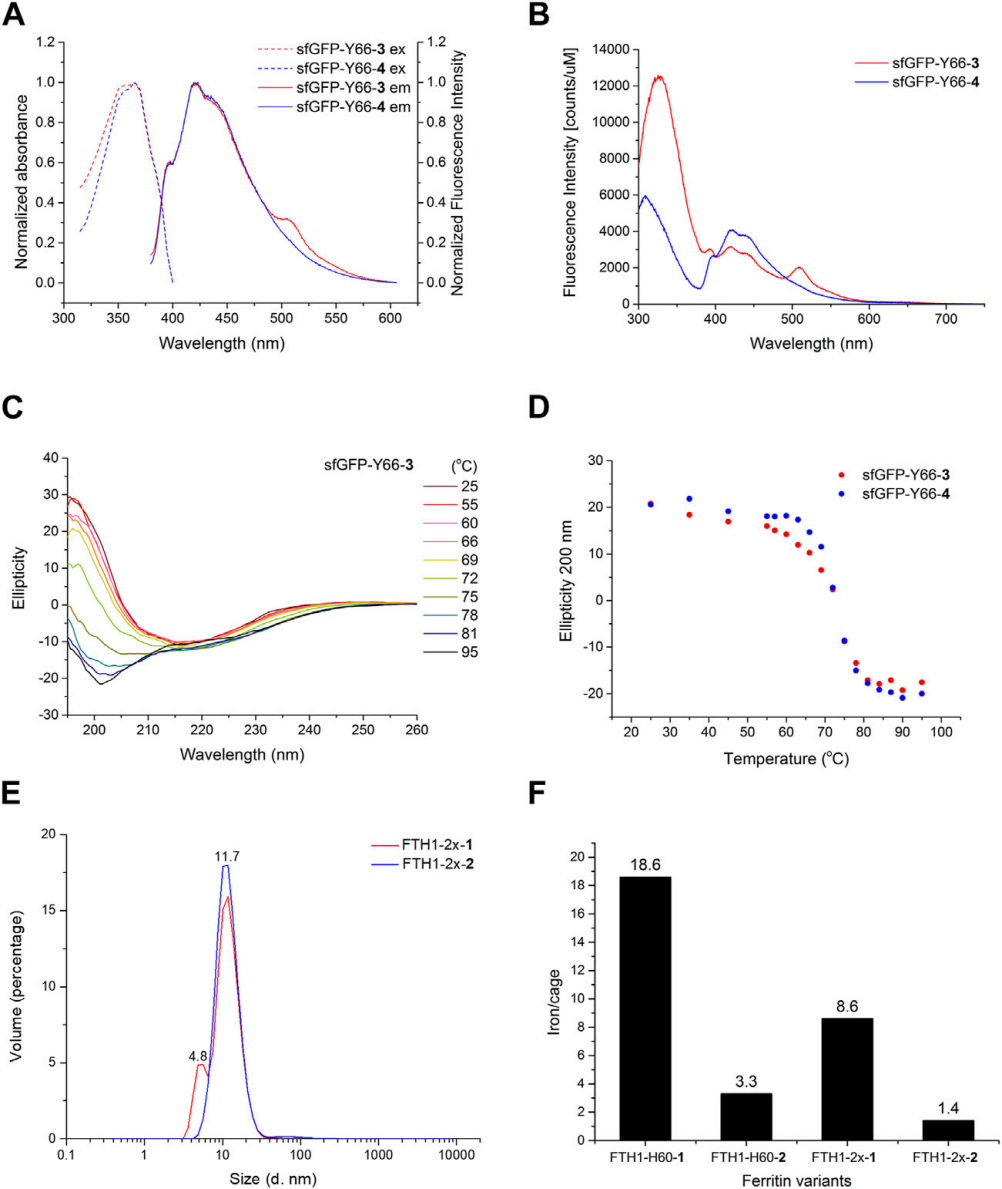
Biophysical analysis of DFA encoded proteins. **(A)** Excitation and emission spectra of sfGFP-Y66-**3** (red) and sfGFP-Y66-**4** (blue). **(B)** Intrinsic fluorescence spectra of sfGFP-Y66-**3** (red) and sfGFP-Y66-**4** (blue). **(C)** Temperature course of ellipticity for sfGFP-Y66-**3** monitored by CD spectroscopy. **(D)** Temperature course of ellipticity at 200 nm for sfGFP-Y66-**3** (red) and sfGFP-Y66-**4** (blue) monitored by CD spectroscopy. **(E)** DLS analysis of FTH1-2x-**1** (red) and FTH1-2x-**2** (blue). **(F)** ICP-MS analysis of FTH1-H60 and FTH1-2x variants. Labeled numbers on the top of each bar indicate the chelated iron per FTH1 molecule.

The intrinsic fluorescence spectrum of wild-type sfGFP showed an intense emission peaking at 509 nm, suggesting that the occurrence of fluorescence resonance energy transfer (FRET) between W57 and surrounding residues can indirectly excite the sfGFP chromophore (Supplementary Figure S7D). With an excitation wavelength of 280 nm, sfGFP-Y66-**3** showed an emission maximum at 330 nm and red-shifted peaks at 420 and 508 nm with much lower intensities, whereas that of sfGFP-Y66-**4** shows a more blue-shifted emission peak maximum at 420 nm (Figure 3B). The variant chromophores of sfGFP-Y66-**3** and **4** may go through FRET pathways (Supplementary Figure S8) upon excitation at 280 nm at W57 (310–330 nm emission), but with less FRET efficiency in comparison to wild-type sfGFP (Supplementary Figure S7D, E). Interestingly, DFA **3**-substituted sfGFP chromophore also showed much lower-level FRET although additional emission peaking around 510 nm was found (Figure 3B), while other sfGFP-Y66 variants showed no significant differences (Supplementary Figure S7E, F).

Given the fact that sfGFP-Y66-**3** and **4** displayed independent emission spectrums, we postulate that these analogs with different chiral orientation in the chromophore might affect the folding and thermal stability of sfGFP. Thus, the conformational stabilities of sfGFP-Y66-**3** and **4** were examined through a temperature course (25°C–95°C) of ellipticity ranging from 195–260 nm, monitored by circular dichroism (CD) spectroscopy (Figure 3C; Supplementary Figure S9A, B). The ellipticity of both sfGFP-Y66-**3** and **4** showed a signature change at 200 nm (Figure 3D). The measured melting temperatures (T_m_) of sfGFP-Y66-**3** and **4** obtained from CD spectra at 205 nm were 73.7°C and 75.5°C, respectively (Supplementary Figure S9C). The ∆H and standard ∆G of sfGFP-Y66-**3** is less by 4.3 and 1.4 kcal mol^-1^, respectively, in comparison to those of sfGFP-Y66-**4**.

### 3.5 Probing the *C*_2_ interface of FTH1 with D/LFAs

Moreover, D/LFAs were introduced to the symmetrical *C*_2_ interface of FTH1, so to manipulate its capability in iron chelating. FTH1, a self-assembled iron-transport protein nanocage, is composed of 24 subunits, in which its heavy chain contains ferroxidases at assembling junction sites (Uchida et al., 2006; Liang et al., 2014). Mutations at residues L56, H60, R63 and E67 of the *C*_2_ interface of FTH1 (Supplementary Figure S10A) are known to disrupt its assembly, causing dissociation of the cage (Huard et al., 2013). H60 and E67 maintain direct interactions that may influence dimer formation in FTH1 early self-assembly process from polar into hydrophobic interactions, then disrupt surface iron binding microenvironment in *C3* and *C4* interfaces. By incorporating D/LFAs at its *C*_*2*_ interface, we attempted to examine if there are any disruptions in iron coordination arrangement at the *C*_*3*_ and *C*_*4*_ symmetric interfaces. ncAAs **1** and **2** were chosen to be incorporated into FTH1 at either position H60 (FTH1-H60-**1** and FTH1-H60-**2**), or positions H60/E67 (FTH1-2x-**1** and FTH1-2x-**2**) through suppression of either one or two UAG codons using the DFRS2·tRNA^Pyl^ pair. Protein masses were then characterized by ESI-MS after purification, resembling the calculated masses (Supplementary Figure S11). The topologies of **1** and **2**-incorporated FTH1 were evaluated by TEM analyses. The TEM images show that these FTH1 variants remain as self-assembled nanocages, revealing that the replacement of FTH1 at positions H60 or H60/E67 with **1** or **2** does not disrupt its assembly (Supplementary Figure S10B–F). Dynamic light scattering (DLS) analyses were also conducted to probe its assembly behavior. We found from particle distribution that FTH1-2x-**2** is dominated by a single species of 11.7 nm in diameter (Figure 3E). Contrastingly, the particle distribution of FTH1-2x-**1** shows that there might be two species present: a major form with a size of 11.7 nm in diameter, and a minor form with a size of 4.8 nm in diameter. Nevertheless, the latter species was not observed during TEM analysis (Supplementary Figure S10B). To determine whether the iron-binding capacity of FTH1 is affected by introducing **1** or **2** at *C*_2_ interface, the numbers of bound iron(III) on FTH1-**1**-**2** variants were analyzed using inductively coupled plasma mass spectrometry (ICP-MS). The results indicated that each FTH1-H60-**1** cage is capable of chelating 6 times more iron atoms than FTH1-H60-**2** (Figure 3F). A similar pattern was observed in FTH1-2x-**1** and FTH1-2x-**2**, although the numbers of bound iron(III) were somehow less than those in FTH1-**1**-**2**. These results suggest that the conformation of the iron binding site of **1** might be distorted, resulting in the enhanced iron chelation compared to **2**.

## 4 Discussion

In summary, we have demonstrated the abilities of DFRS1 and DFRS2 in charging their cognate tRNA^Pyl^ with both D/LFAs *in vivo*. Equipped with the R61K/H63Y/S193R mutations, we found DFRS2 to be more catalytically efficient than DFRS1 in the presence of D/LFAs. Despite DFRS2 is capable of aminoacylating the tRNA^Pyl^ with both D- and LFAs, as expected, the enzyme still preferentially recognizes LFAs over DFAs as substrates according to kinetics data. This observation agrees well with data obtained from *in vivo* amber suppression assays. Similar with reported PylRS-N346A/C348A, LFRS and DFRS2 show rarely LPhe incorporation from nineteen LAAs and DAAs. It is noted that the more soluble C-terminal domain of PylRS is less active *in vitro*, but shows no activity *in vivo* compared to the full-length enzyme (Herring et al., 2007). We interrogated the activity of purified DFRSc on amino acid activation using D/LFAs. Although DFRSc activity was detected in the presence of LFAs, the enzyme showed no activity when DFAs were added to the reaction mixtures. Based on our kinetics data, we assume that DAAs might not adopt the typical two-step reaction of LAAs. However, we acknowledge that the solubility of DFAs presented challenges in producing comprehensive amino acid activation kinetics for the mutant enzyme. This limitation prevented us from obtaining direct kinetic parameters for DFAs in the same manner as for LFAs. The truncated enzyme may hold a high *K*_*M*_ for DFAs, being beyond the solubility limits of these ncAAs. The active-site N346G/C348Q/V401G mutations of DFRS2 contribute to the enlarging of its active site, which leads to insufficient *K*_*M*_ in charging ncAAs, as demonstrated by the outcomes of ATP-PP_i_ experiments with DFRSc.

Aminoacylation assays of DFRS2 against LFAs and DFAs, however, indicate the overall outcome of both half-reactions (i.e., the former being amino acid activation and the latter being aminoacylation). Results show detectable activity between DFRS2 and all DFAs, and even higher activities between DFRS2 and LFAs in comparison with the PylRS in acylating BocK. This result further suggests that both the assistance of the N-terminal domain and tRNA binding are essential for ncAA accommodation, thereby also contributing to the following ncAA activation and acylation steps, synchronously and significantly at DFRS2’s active site. Based on *in vivo* amber suppression assays and kinetic studies of DFRS2, the R61K/H63Y/S193R mutations at DFRS2’s N-terminal domain are shown to enhance its binding to tRNA’s T-arm and T-loop regions, and to remotely tighten up its active pocket for better ncAA-activation and acylation. Furthermore, crystal structures of DFA·DFRSc complexes have revealed a clear but more dynamic binding mode than those of LFA·DFRSc complexes. These structures suggest that DFRS2’s active site binds loosely to amino acid main chains when recognizing DFAs, which may be allowing the aminoacylation of tRNA^Pyl^ with DFAs. The additional V401G mutation of DFRS2 generates an enlarged pocket, making DFRS2 more capable of recognizing both D- and LFAs as substrates in comparison with the PylRS N346G/C348Q variant. The wobbling of DFAs in its active site may explain why the DFA-activating activity of DFRSc was undetectable.

D-amino acids have long been implemented to generate optically active peptides and proteins for therapeutic purposes or novel protein design. By adding D-ncAAs into the toolbox of protein engineering, this work expands the scope of ribosomal protein synthesis using an evolved PylRS·tRNA^Pyl^ pair. Genetically encoded D-ncAAs can be used as versatile tools other than L-ncAAs to modify protein with new properties or for *de novo* protein design. In this study, we have shown that, by introducing DFA **3** and LFA **4**, even a single-residue change in the chirality of the sfGFP chromophore can affect its biophysical characteristics, including those seen in its emission spectrum and thermal stability. Compared with its L-form counterpart sfGFP-Y66-**4**, sfGFP-Y66-**3** displays an extra peak in the emission spectrum, resulting in 1.8°C and 1.4 kcal mol^-1^ deductions in Tm and standard ∆G values, respectively. These thermodynamics differences reveal that the chromophore’s spatial arrangements featuring D-chirality are more destabilizing than those of its L-form analogs during temperature progression. This observation indicates that the thermal stability of proteins can be affected by swapping the chirality of ncAAs, even with a single residue change. Despite sharing similar particle size, incorporation of DFA **1** at the *C*_2_ interface of FTH1 improved its iron-binding capacity by six folds compared to that of the LFA **2**-encoded variant.

In contrast, DFA **1** incorporation at the *C*_2_ interface of FTH1 shows higher efficiency in iron coordination while the assembled structure of FTH1 remains intact. The dimer form of FTH1 is the primary component in its self-assembling process (Theil, 2011; Huard et al., 2013). The remodeled *C*_2_ interfaces of FTH1 with DFA **1** and LFA **2** demonstrated that single residue chirality mutations can significantly change the molecular microenvironment in metal chelating. This discovery again demonstrates the use of DFAs in expanding the chemical and chiral diversity of building blocks for protein engineering. Taken together, this study broadens the toolbox of genetic code expansion by creating a PylRS·tRNA^Pyl^ system that can site-selectively incorporate LFAs and DFAs into proteins in *E. coli* cells.

## Data Availability

The datasets presented in this study can be found in online repositories. The names of the repository/repositories and accession number(s) can be found in the article/Supplementary Material.
