# Supplementary material for: Rational design of the genetic code expansion toolkit for in vivo encoding of D-amino acids
Source: Front Genet. 2023 Oct 13;14:1277489. doi: 10.3389/fgene.2023.1277489 (PMC10613524; doi:10.3389/fgene.2023.1277489)
Supplement: Supplementary file 1 [file DataSheet4.pdf]

Supporting Information For:

Rational design of the genetic code expansion toolkit for in vivo encoding of D-amino acids

Han-Kai Jiang[1, 2, 3†], Jui-Hung Weng[1†], Yi-Hui Wang[1, 4†], Jo-Chu Tsou[1], Pei-Jung Chen[1, 4], An-Li Andrea Ko[1], Dieter Söll[5, 6], Ming-Daw Tsai[1], and Yane-Shih Wang[1, 2, 4*]

[1]Institute of Biological Chemistry, [2]Chemical Biology & Molecular Biophysics Program Taiwan International Graduate Program, Academia Sinica, Taipei, Taiwan

[3]Department of Chemistry, National Tsing Hua University, Hsinchu, Taiwan

[4]Institute of Biochemical Sciences, National Taiwan University, Taipei, Taiwan

[5]Department of Molecular Biophysics & Biochemistry, [6]Department of Chemistry, Yale University, New Haven, CT, USA

[†]These authors have contributed equally to this work and share first authorship.

*Correspondence should be addressed to Yane-Shih Wang (yaneshihwang@gate.sinica.edu.tw).

1. DNA and protein sequences

1.1 DNA sequence

$tRNA^{Pyl}_{CUA}$:

ggaaacctgatcatgtagatcgaatggactctaaatccgttcagccgggttagattcccggggtttccgcca

Methanosarcina mazei PylRS:

atggataaaaaaccactaaacactctgatatctgcaaccgggctctggatgtccaggaccggaacaattcataaaataa
aacaccacgaagtctctcgaagcaaaatctatattgaaatggcatgcggagaccaccttgttgtaaacaactccaggagc
agcaggactgcaagagcgctcaggcaccacaaatacaggaagacctgcaaacgctgcagggtttcggatgaggatct
caataagttcctcacaaaggcaaacgaagaccagacaagcgtaaaagtcaaggtcgtttctgcccctaccagaacgaa
aaaggcaatgccaaaatccgttgcgagagccccgaaacctcttgagaatacagaagcggcacaggctcaaccttctgg
atctaaattttcacctgcgataccggtttccacccaagagtcagtttctgtcccggcatctgtttcaacatcaatatcaagcatt
tctacaggagcaactgcatccgcactggtaaaagggaatacgaaccccattacatccatgtctgcccctgttcaggcaagt
gcccccgcacttacgaagagccagactgacaggcttgaagtcctgttaaacccaaaagatgagatttccctgaattccgg
caagcctttcagggagcttgagtccgaattgctctctcgcagaaaaaaagacctgcagcagatctacgcggaagaaagg
gagaattatctggggaaactcgagcgtgaaattaccaggttctttgtggacaggggttttctggaaataaaatccccgatcc
tgatccctcttgagtatatcgaaaggatgggcattgataatgataccgaactttcaaaacagatcttcagggttgacaagaa
cttctgcctgagacccatgcttgctccaaacctttacaactacctgcgcaagcttgacagggccctgcctgatccaataaaa
atttttgaaataggcccatgctacagaaaagagtccgacggcaaagaacacctcgaagagtttaccatgctgaacttctg
ccagatgggatcgggatgcacacgggaaaatcttgaaagcataattacggacttcctgaaccacctgggaattgatttca
agatcgtaggcgattcctgcatggtctatggggataccсttgatgtaatgcacggagacctggaactttcctctgcagtagtcggaccca
taccgcttgaccgggaatggggtattgataaaccctggataggggcaggtttcgggctcgaacgccttctaaa
ggttaaacacgactttaaaaatatcaagagagctgcaaggtccgagtcttactataacgggatttctaccaacctgtaa

sfGFP:

atgagcaagggcgaagaactgtttacgggcgtggtgccgattctggtggaactggatggtgatgtcaatggtcacaaattc
agcgtgcgcggcgaaggtgaaggcgatgcaaccaatggtaaactgacgctgaagtttatttgcaccacgggtaaactgc
cggttccgtggccgaccctggtcaccacgctgacgtatggtgttcagtgtttcagtcgttacccggatcacatgaaacgcca
cgactttttcaagtccgcgatgccggaaggttatgtccaagaacgtaccatctcatttaaagatgacggcacctacaaaac
gcgcgccgaagtgaaattcgaaggtgatacgctggttaaccgtattgaactgaaaggcatcgattttaaggaagacggta
atattctgggccataaactggaatataacttcaattcgcacaacgtgtacatcaccgcagataagcagaagaacggtatc
aaggctaacttcaagatccgccataatgtggaagatggcagcgttcaactggccgaccactatcagcaaaacaccccga
ttggtgatggcccggtcctgctgccggacaatcattacctgagcacgcagtctgtgctgagtaaagatccgaacgaaaag

cgtgaccacatggtcctgctggaattcgtgaccgcggccggcatcacgcacggtatggacgaactgtataaaggctcag
agctccatcaccatcaccatcactaa

Human heavy chain ferritin:

atgaccaccgcctctacctcacaggtgcgtcagaattatcatcaggatagtgaagcagcaattaatcgccagattaatctg
gaactgtatgcaagctatgtgtatctgtctatgagctattattttgatcgcgatgatgttgccctgaaaaattttgccaaatatttt
ctgcatcagtctcatgaagaacgcgaacatgccgaaaaactgatgaaattacagaatcagcgtggtggtcgtatttttcttc
aagatattaaaaaaccggattgtgatgattgggaaagcggcctgaatgcgatggaatgtgccttacatcttgagaaaaat
gttaatcagtcactgctggaactgcataaactggcaaccgataaaaatgatccgcatctgtgtgattttattgaaacccatta
tctgaatgagcaggttaaagccattaaagaactgggcgatcatgttaccaatctccgcaaaatgggcgccccggaaagtggcttagcc
gaatatctgtttgataaacataccttaggcgatagcgataacgaaagtgagctccatcaccatcaccatcactaa

1.2 Protein sequence

Methanosarcina mazei PylRS:

MDKKPLNTLISATGLWMSRTGTIHKIKHHEVSRSKIYIEMACGDHLVVNNSRSSRTAR
ALRHHKYRKTCKRCRVSDEDLNKFLTKANEDQTSVKVKVVSAPTRTKKAMPKSVAR
APKPLENTEAAQAQPSGSKFSPAIPVSTQESVSVPASVSTSISSISTGATASALVKGNTNP
ITSMSAPVQASAPALTKSQTDRLEVLLNPKDEISLNSGKPFRELESELLSRRKKDLQQI
YAEERENYLGKLEREITRFFVDRGFLEIKSPILIPLEYIERMGIDNDTELSKQIFRVDKNF
CLRPMLAPNLYNYLRKLDRALPDPIKIFEIGPCYRKESDGKEHLEEFTMLNFCQMGSG
CTRENLESIITDFLNHLGIDFKIVGDSCMVYGDTLDVMHGDLELSSAVVGPIPLDREW
GIDKPWIGAGFGLERLLKVKHDFKN IKRAARSESYYNGISTNL*

sfGFP:

MSKGEELFTGVVPILVELDGDVNGHKF^{27}SVRGEGEGDATNGKLTLKFICTTGKLPVP
WPTLVTTLTYGVQCFSRYPDHMKRHDFFKSAMPEGYVQERTISFKDDGTYKTRAEVK
FEGDTLVNRIELKGIDFKEDGNILGHKLEYNFNSHNVYITADKQKNGIKANFKIRHNV
EDGSVQLADHYQQNTPIGDGPVLLPDNHYLSTQSVLSKDPNEK
RDHMVLLEFVTAAGITHGMDELYKGSELHHHHHH*

The residues colored in red represent the chosen position for ncAA incorporation.

Human heavy chain ferritin:

MTTASTSQVRQNYHQDSEAAINRQINLELYASYVYLSMSYYFDRDDVALKNFAKYFL
HQSHEEREHAEKLMKLQNQRGGRIFLQDIKKPDCDDWESGLNAMECALHLEKNVN
QSLLELHKLATDKNDPHLCDFIETHYLNEQVKAIKELGDHVTNLRKMGAPESGLAEY

LFDKHTLGDSDNESELHHHHHH*

2. Plasmid construction

2.1. Primer List

Name	Sequences(5' to 3')
pET-h-Ferritin-NdeI-F	gatatacatatgaccaccgcctctacctcacaggtgc
pET-h-Ferritin-SacI-R	ggtgatggagctcactttcgttatcgctatcgcctaag
PylRS-c270-F2	cacttacgaagagccagactgacaggcttg
pET-PylRS-R2	ggtgatggagctcttacaggttggtagaaatcccg
pET-His6X-F1	gagatatacatatgcatcaccatcaccatcac
His6x-PylRS185-R1	ctcttcgtaagtgcgggggcacttgcgtgatggtgatgg
pET-sfGFP-NdeI-F1	gagatatacatatgagcaagggcgaag
pET-sfGFP-SacI-R2	gatggtgatggagctctgagcctttatac
sfGFP-Y66TAG-F2	gtcaccacgctgacgtagggtgttcag
sfGFP-Y66TAG-R1	ctgaacaccctacgtcagcgtggtgac
pET-sfGFP-F27TAG-R	caccttcgccgcgcacgctctatttgtgacc
pET-sfGFP-F27TAG-F	ggtcacaaatagagcgtgcgcggcgaaggtg
h-Ferritin-H60TAG-F	ctgcatcagtcttaggaagaacgcgaacatg
h-Ferritin-H60TAG-R	catgttcgcgttcttcctaagactgatgcag
h-Ferritin-E67TAG-F	cgcgaacatgcctagaaactgatgaaattac
h-Ferritin-E67TAG-R	gtaatttcatcagtttctaggcatgttcgcgttc
pCDF-PylRS-NcoI-F	gtattaaccatggataaaaaaccactaaac
pCDF-PylRS-EcoRI-F	gatttccctgaattccggcaagcctttcagggagc
pCDF-PylRS-EcoRI-R	ggcttgccggaattcagggaaatctcatcttttg
PylRS-R61K/H63Y-F	gcaggactgcaagagcgctcaaacactataaatacaggaag
PylRS-R61K/H63Y-R	tttgcaggtcttcctgtatttatagtgtttgagcgctcttg
PylRS-S193R-F	caggcaagtgcccccgcacttacgaagcgtcagactgacag
PylRS-S193R-R	gacttcaagcctgtcagtctgacgcttcgtaagtgcg
PylRS-N346V-F	ccatgctggtgttctgccagatggg
PylRS-N346V-R	cccatctggcagaacaccagcatgg
PylRS-N346G/C348Q-F	ccatgctgggcttccagcagatgggatc
PylRS-N346G/C348Q-R	gatcccatctgctggaagcccagcatgg
pCDF-PylRS-V401G-F	cctggaactttcctctgcaggcgtcggacccataccgcttgacc
pCDF-PylRS-V401G-R	ggtcaagcggtatgggtccgacgcctgcagaggaaagttccagg

pCDF-PylRS-BamHI-R	ggtcgacggatccttacaggttggtagaaatcccgttatag

2.2. Construction of pCDF-*PylRS* variants

PCR was performed using the KOD hot start polymerase kit (Merck). Oligonucleotide synthesis and DNA sequencing were done by Genomics Inc. (Taipei, Taiwan). Synthesized *MmPylRS* gene was subcloned into pCDF-1b plasmid using restriction enzymes NcoI at 5' end and EcoRI at 3' end. General protocol for constructing different PylRS variants followed the same procedure described below. The mutations were generated by overlap extension PCR from pCDF-*MmPylRS*. The following primer pairs were used to yield *nPylRS* gene: (1) pCDF-PylRS-NcoI-F and PylRS-R61K/H63Y-R; (2) PylRS-R61K/H63Y-F and pCDF-PylRS-BamHI-R; (3) pCDF-PylRS-NcoI-F and PylRS-S193R-R; (4) PylRS-S193R-F and pCDF-PylRS-BamHI-R. The amplified *nPylRS* gene was double digested with restriction enzymes NcoI and EcoRI followed by gel purification and ligation to generate pCDF-*nPylRS*. The following primer pairs were used to yield *LFRS* gene: (1) pCDF-PylRS-EcoRI-F and PylRS-N346V-R; (2) PylRS-N346V-F and pCDF-PylRS-BamHI-R. Double digested and gel-purified *LFRS* gene then ligated back into pCDF vector to yield pCDF-*LFRS*. The following primer pairs were used to yield *DFRS1* gene: (1) pCDF-PylRS-EcoRI-F and PylRS-N346G/C348Q-R; (2) PylRS-N346G/C348Q-F and pCDF-PylRS-BamHI-R; (3) pCDF-PylRS-EcoRI-F and pCDF-PylRS-V401G-R; (4) pCDF-PylRS-V401G-F and pCDF-PylRS-BamHI-R. Double digested and gel-purified *DFRS1* gene then ligated back into pCDF vector to yield pCDF-*DFRS1*. To construct pCDF-*DFRS2*, the mutations were introduced by overlap extension PCR from pCDF-*nPylRS* and pCDF-*DFRS1*. The following primer pairs were used to yield *DFRS2* gene: (1) pCDF-PylRS-NcoI-F and pCDF-PylRS-EcoRI-R; (2) pCDF-PylRS-EcoRI-F and pCDF-PylRS-BamHI-R. Double digested and gel-purified *DFRS2* gene then ligated back into pCDF vector to yield pCDF-*DFRS2*.

2.3. Construction of pET-*pylT-sfGFP* variants

Synthesized *Methanosarcina mazei* pyrrolysyl-tRNA gene (*pylT*) was subcloned into pET22b (+) plasmid using restriction enzymes NcoI at 5' end and PstI at 3' end to yield pET-*pylT*. The *sfGFP* gene was synthesized and subcloned into pET-*pylT* plasmid using restriction enzymes NdeI at 5' end and SacI *at* 3' end to generate pET-*pylT-sfGFP*. Four positions (F27 and Y66) in *sfGFP* gene were mutated to amber codons by overlap extension PCR from pET-*pylT-sfGFP*, respectively. The following pairs of primer were used to generate an amber mutation in *sfGFP* gene: (1) pET-sfGFP-NdeI-F1 and pET-sfGFP-F27TAG-R; pET-sfGFP-F27TAG-F and pET-sfGFP-SacI-R2; (2) pET-sfGFP-NdeI-F1 and sfGFP-Y66TAG-R1; sfGFP-

Y66TAG-F2 and pET-sfGFP-SacI-R2. The amplified genes (*sfGFP-F27TAG* and *sfGFP-Y66TAG*,) were double digested with NdeI and SacI-HF, gel-purified and ligated back into pET-*pylT* vector respectively. All sfGFP variants contain hexahistidine tag at C-terminus.

2.4. Construction of pET-*pylT-FTH1* variants

The pET-*pylT-FTH1* plasmid was derived from pET-*pylT*. Synthesized human heavy chain ferritin *(Fer)*gene carrying C-terminal hexahistidine tag was subcloned into pET-*pylT* using restriction enzymes NdeI at 5' end and SacI at 3' end to generate pET-*pylT-FTH1*. Ferritin variants Fer-H60 bearing an amber codon, pET-*FTH1-H60*, was generated using overlap extension PCR from pET-*pylT-FTH1*. The following primer pairs were used to generate an amber mutation in *Fer* gene: pET-h-Ferritin-NdeI-F and h-Ferritin-H60TAG-R; h-Ferritin-H60TAG-F and pET-h-Ferritin-SacI-R. The amplified products were double digested with NdeI and SacI-HF, gel-purified and ligated back into pET-*pylT* vector to generate pET-*pylT-FTH1* variants. To construct pET-*FTH1-2xTAG*, the mutations were introduced by overlap extension PCR from pET-*FTH1-H60*. The following primer pairs were used to yield *Fer-H60* gene: pET-h-Ferritin-NdeI-F and h-Ferritin-H60/E67TAG-R; h-Ferritin-H60/E67TAG-F and pET-h-Ferritin-SacI-R. The amplified product was double digested with NdeI and SacI-HF, gel-purified and ligated back into pET-*pylT* vector.

2.5. Construction of pET-*pylT-DFRSc*

Plasmid pET-*DFRSc*, carrying the gene encoding C-terminal domain of DFRS2$_{185\text{-}454}$ (~270 residues) with N-terminal hexahistidine tag, was derived from the plasmid pCDF- *DFRS2*. The following pairs of primer were used to generate *DFRS2c* gene: (1) pET-His6X-F1 and His6x-PylRS185-R1; (2) PylRS-c270-F2 and pET-PylRS-R2. The amplified product was double digested with NdeI and SacI, gel-purified and ligated back into pET22b (+) vector to generate pET-*DFRSc*.

Table S1. Mutation sites of PylRS variants in this study.

PylRS	R61	H63	S193	N346	C348	V401
nPylRS	K	Y	R			
LFRS				V		
DFRS1				G	Q	G
DFRS2	K	Y	R	G	Q	G

Table S2. Data collection and refinement statistics.

ncAA	DFF (**1**)	LFF (**2**)	DCF (**3**)	LCF (**4**)	DBF (**5**)	LBF (**6**)
PDB ID	8KE3	8KE2	8KE5	8KE6	8KE4	8KE1
**Data collection**	NSRRC 15A	NSRRC 15A	NSRRC 15A	NSRRC 15A	NSRRC 15A	NSRRC 15A
Resolution (Å)	33.4 - 1.9	24.8 - 2.2	34.3 - 1.8	26.3 - 1.9	28.0 - 1.8	28.0 - 2.5
Space group	$P6_4$	$P6_4$	$P6_4$	$P6_4$	$P6_4$	$P6_4$
Cell dimensions						
a, *b*, *c* (Å)	104.67	105.508	104.69	105.16	105.20	104.96
	104.67	105.508	104.69	105.16	105.20	104.96
	71.813	71.13	71.34	72.45	71.69	71.11
Wavelength (Å)	1.0000	1.0000	1.0000	1.0000	1.0000	1.0000
No. unique reflections	34993	22987	41612	36108	45459	15531
R_{merge}	0.057	0.078	0.048	0.049	0.048	0.060
$I / \Sigma i$	21.4	19.7	34.1	42.8	55.5	39.7
Completeness (%)	98.9 (99.3)	99.6 (100)	99.9(99.9)	99.7 (100)	100 (100)	100 (100)
Redundancy	6.9 (6.3)	6.8 (6.9)	9.8(9.3)	7.3 (7.4)	11.4 (11.3)	7.6 (7.7)
**Refinement**						
Resolution (Å)	33.4 - 1.9	24.8 - 2.2	34.3 - 1.9	26.3 - 1.9	28.0 - 1.8	28.0 - 2.5
Reflections (working/test)	33828 / 1999	22457 / 1992	35140 / 1719	36108 / 1987	45459 / 2009	15512 / 1531
R_{work} / R_{free}	0.188 / 0.211	0.190 / 0.221	0.184 / 0.186	0.173 / 0.189	0.169 / 0.182	0.175 / 0.201
R.m.s deviations						
Bond lengths (Å)	0.012	0.013	0.010	0.008	0.011	0.008
Bond angles (°)	1.17	1.28	1.300	0.954	1.07	1.11
No. atoms / *B*-factors						
Protein	2133 / 24.7	2094 / 37.1	2108 / 23.6	2118 / 35.0	2114 / 29.08	2113 / 37.1
Water	117 / 26.4	126 / 37.3	145 / 27.24	207/ 40.78	295 / 39.77	84 / 38.6
Ligand	49 / 21.6	49 / 33.0	46 / 20.4	46 / 32.06	46 / 25.73	46 / 34.4
Ramachandran plot						
Favored %	97.65	98.43	98.04	97.66	98.06	98.05
Allowed %	2.35	1.57	1.96	2.34	1.94	1.95
Outliers %	0.00	0.00	0.00	0.00	0.00	0.00
Rotamer outliers %	0.00	0.00	0.00	0.00	0.00	0.00

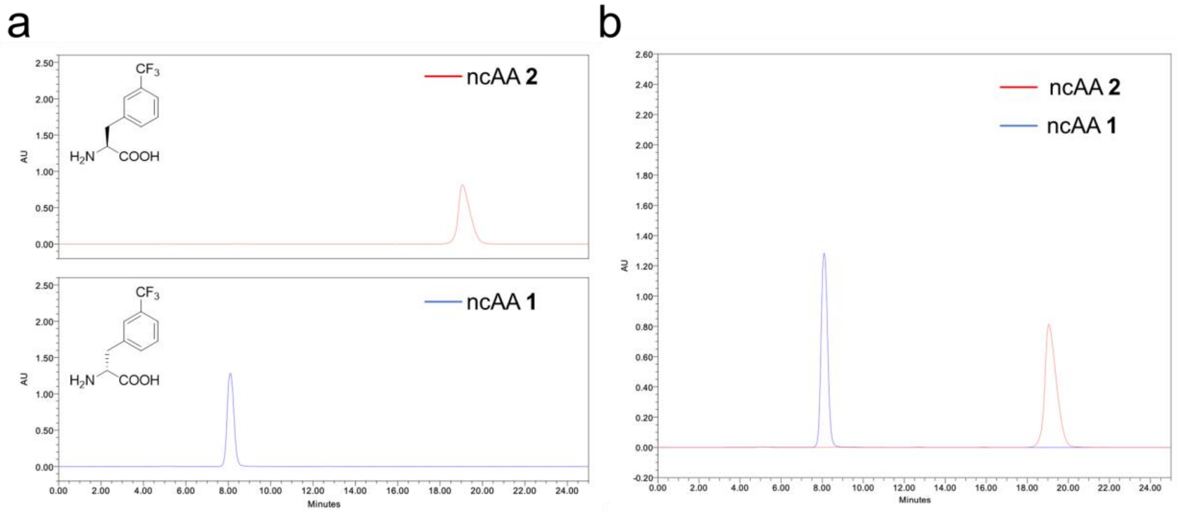

Figure S1. Chiral HPLC analysis of ncAA 1 and 2.

(a) Determination of chiral purity of ncAA **1** (blue) and **2** (red). (b) Overlapping HPLC profiles of ncAA **1** (blue) and **2** (red). Chromatographic conditions: CROWNPAKS CR-I (+) (3 mm × 150 mm, 5 μm particle size); aqueous solution of $HClO_4$ (pH 1.5) / acetonitrile = 70 : 30 (v/v) was used as mobile phase at a flow rate of 0.2 mL/min with UV detection at 200 nm at 25 °C.

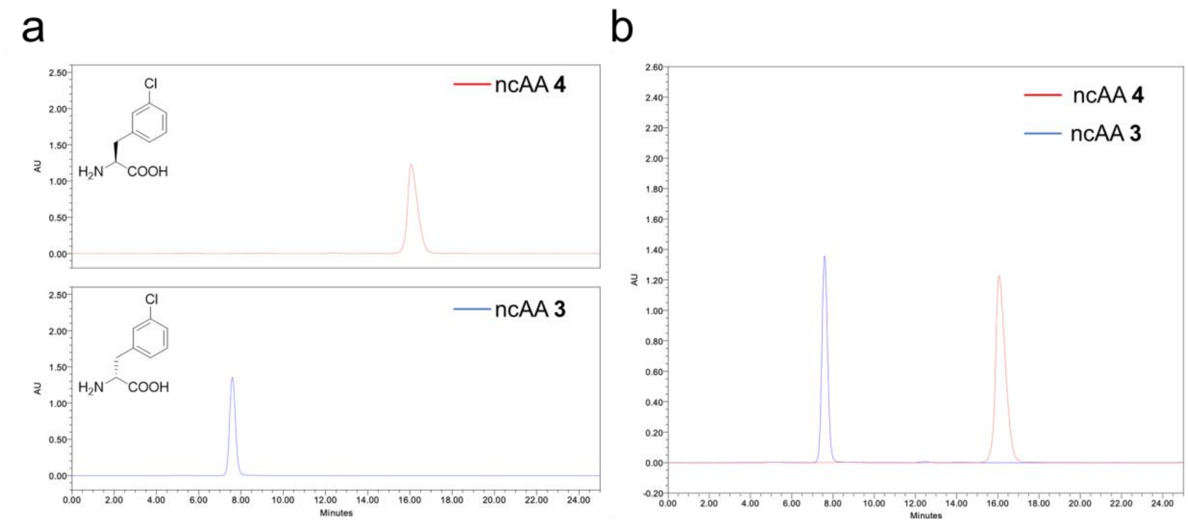

Figure S2. Chiral HPLC analysis of ncAA 3 and 4.

(a) Determination of chiral purity of ncAA **3** (blue) and **4** (red). (b) Overlapping HPLC profiles of ncAA **3** (blue) and **4** (red). Chromatographic conditions: CROWNPAKS CR-I (+) (3 mm × 150 mm, 5 μm particle size); aqueous solution of $HClO_4$ (pH 1.5) / acetonitrile = 70 : 30 (v/v) was used as mobile phase at a flow rate of 0.2 mL/min with UV detection at 200 nm at 25 °C.

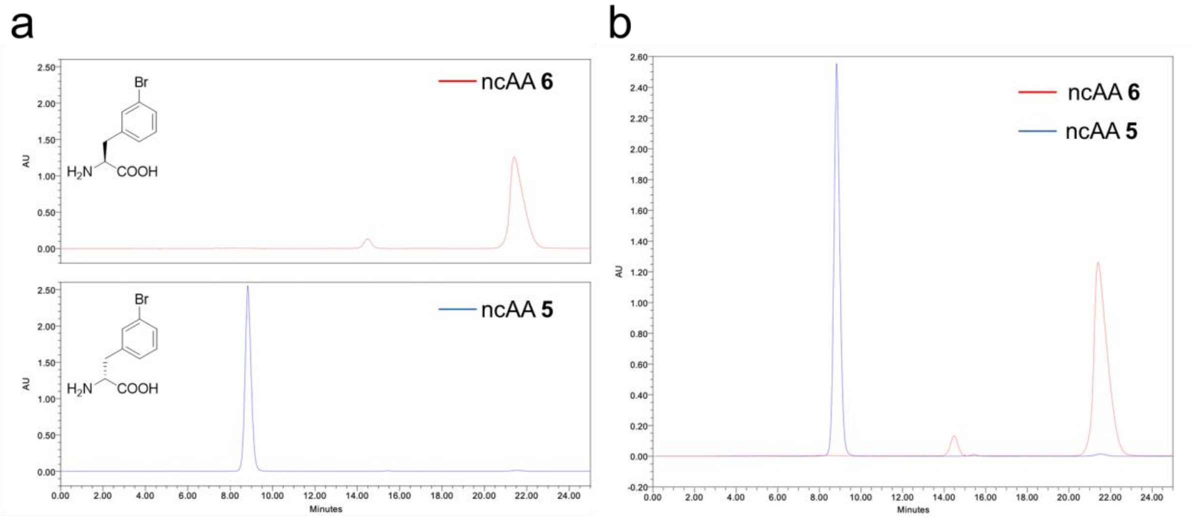

Figure S3. Chiral HPLC analysis of ncAA 5 and 6.

(a) Determination of chiral purity of ncAA **5** (blue) and **6** (red). (b) Overlapping HPLC profiles of ncAA **5** (blue) and **6** (red). Chromatographic conditions: CROWNPAKS CR-I (+) (3 mm × 150 mm, 5 μm particle size); aqueous solution of $HClO_4$ (pH 1.5) / acetonitrile = 70 : 30 (v/v) was used as mobile phase at a flow rate of 0.2 mL/min with UV detection at 200 nm at 25 °C.

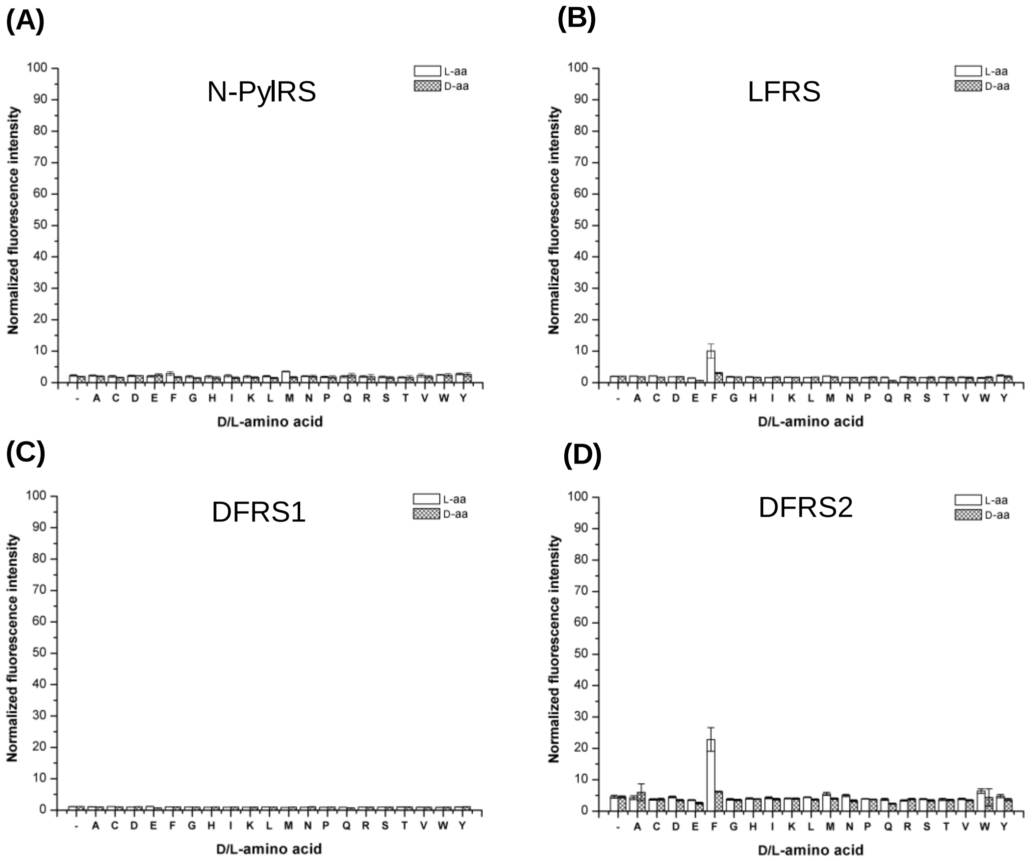

Figure S4. Incorporation efficiency of PylRS mutants with nineteen DAA and LAA. Suppression efficiency of PylRS variants, (A) N-PylRS; (B) LFRS; (C) DFRS1; (D) DFRS2, was examined using a sfGFP-27TAG reporter in *E. coli* BL21 (DE3) cells in GMML medium containing a dedicated ncAA. UAG translation efficiency was obtained by normalizing the fluorescence of sfGFP-27TAG to wild-type sfGFP (the signal of wild-type sfGFP is viewed 100%). Data are presented the mean ± SD for four replicates. The LAA (white bar) and DAA (cross bar) with nineteen LAA/DAA pairs are listed with one letter abbreviation. The G indicates glycine, which has no chiral center.

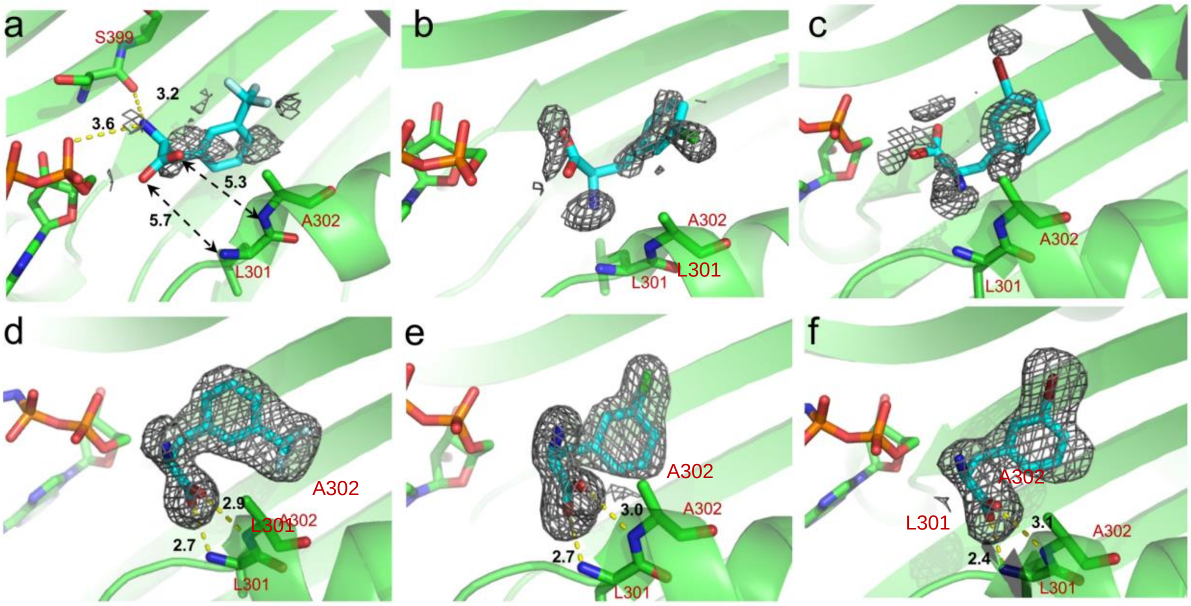

Figure S5. Electron density map (*F*o-*F*c) around D/LFA in DFRSc binding pocket calculated from the D/LFA omitted structure. Green cartoon structure represents DFRSc in complex with (a) **1**, (b) **3**, and (c) **5** (d) **2**, (e) **4**, and (f) **6** (σ = 3.0). All LFAs (**2**, **4**, and **6**) can be built and fitted well to the electron density. In contrast, only partial electron density can be identified for the DFAs (**1**, **3**, and **5**). Hydrogen bonds are shown in yellow dashed lines.

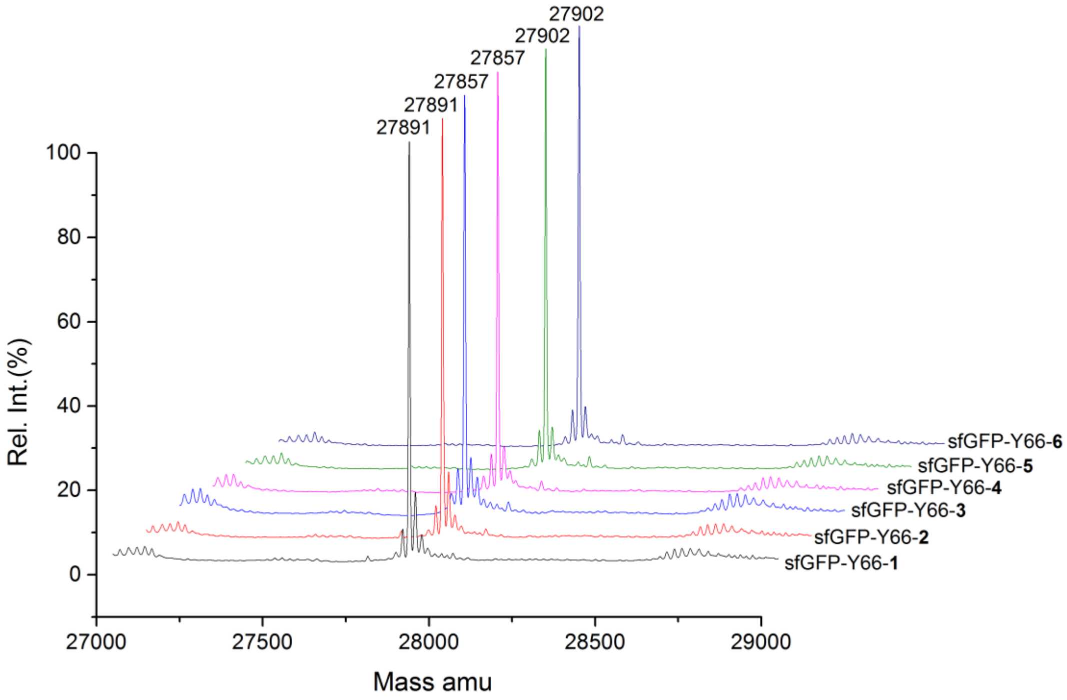

Figure S6. ESI-MS analysis of sfGFP-Y66 variants.

Full-length sfGFP-Y66 variants were produced in *E. coli* BL21(DE3) cells that were co-expressing sfGFP-66TAG gene and the DFRS2·$tRNA^{Pyl}$ pair with the supplement of 1 mM **1-6** in GMML medium at 37 ºC for 12 h. The calculated masses of sfGFP-Y66-**1**(or -**2**), sfGFP-Y66-**3** (or -**4**), and sfGFP-Y66-**5** (or -**6**) are 27,891 Da (–Met), 27,857 Da (–Met), and 27,902 Da (–Met). The observed masses of these sfGFP-Y66 variants match the calculated masses well.

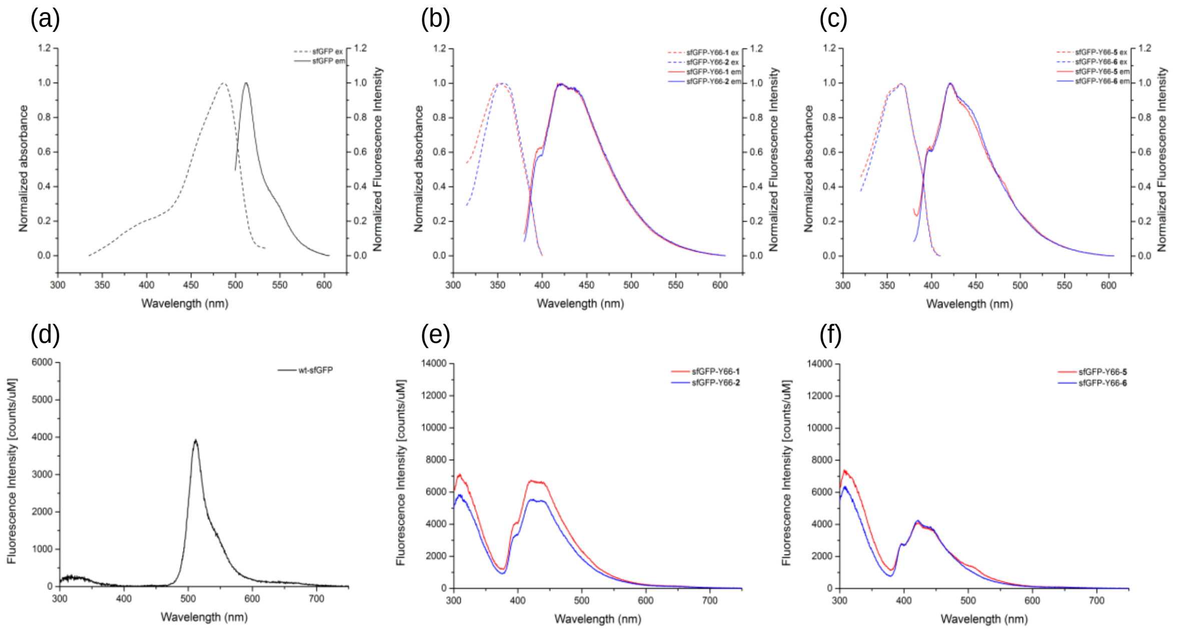

Figure S7. Spectral analysis of sfGFP-Y66 variants.

Excitation and emission spectra of (a) wild-type sfGFP (ex. 485 nm, em. 511 nm). Wild-type sfGFP exhibited a 26 nm Stokes shift. (b) sfGFP-Y66-**1** (red) and sfGFP-Y66-**2** (blue) (ex. 350 nm, em. 420 nm). Both sfGFP-Y66-**1** and **2** showed 70 mm Stokes shifts. (c) sfGFP-Y66-**5** (red) and sfGFP-Y66-**6** (blue) (ex. 365 nm, em. 420 nm). Both sfGFP-Y66-**5** and **6** displayed 55 nm Stokes shifts. The excitation and emission spectra are shown in dashed and solid lines. Intrinsic fluorescence spectra of (d) wild-type sfGFP (ex. 280 nm, em. 322 and 509 nm). (e) sfGFP-Y66-**1** (red) and sfGFP-Y66-**2** (blue) (ex. 280 nm, em. 322 and 420 nm). (f) sfGFP-Y66-**5** (red) and sfGFP-Y66-**6** (blue) (ex. 280 nm, em. 322 and 420 nm).

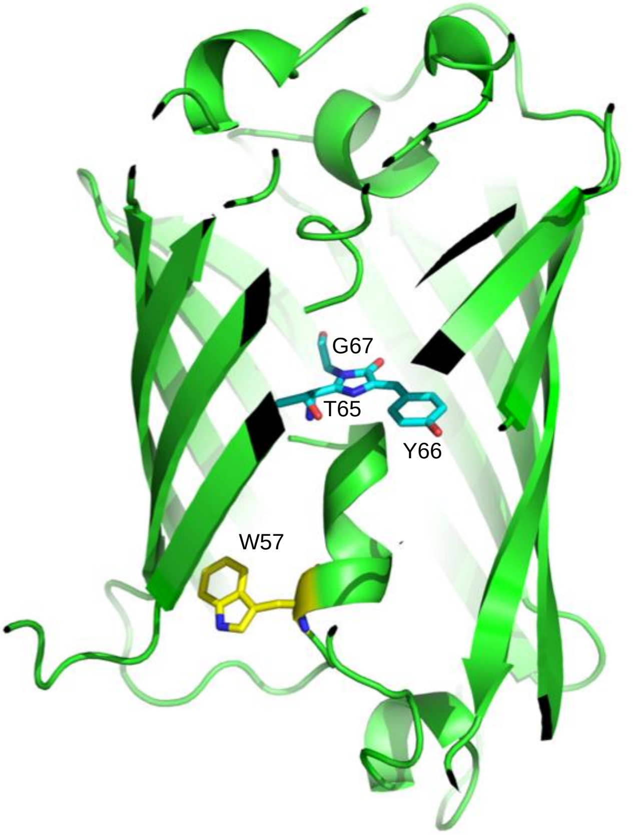

Figure S8. Chromophore core of superfolder green fluorescent protein.

The cyclic chromophore, T65-Y66-G67, of sfGFP is shown as a cyan stick (PDB code: 2B3P). The only tryptophan 57 (W57) of sfGFP is labelled in yellow stick. The distance between chromophore and W57 is around 17 Å.

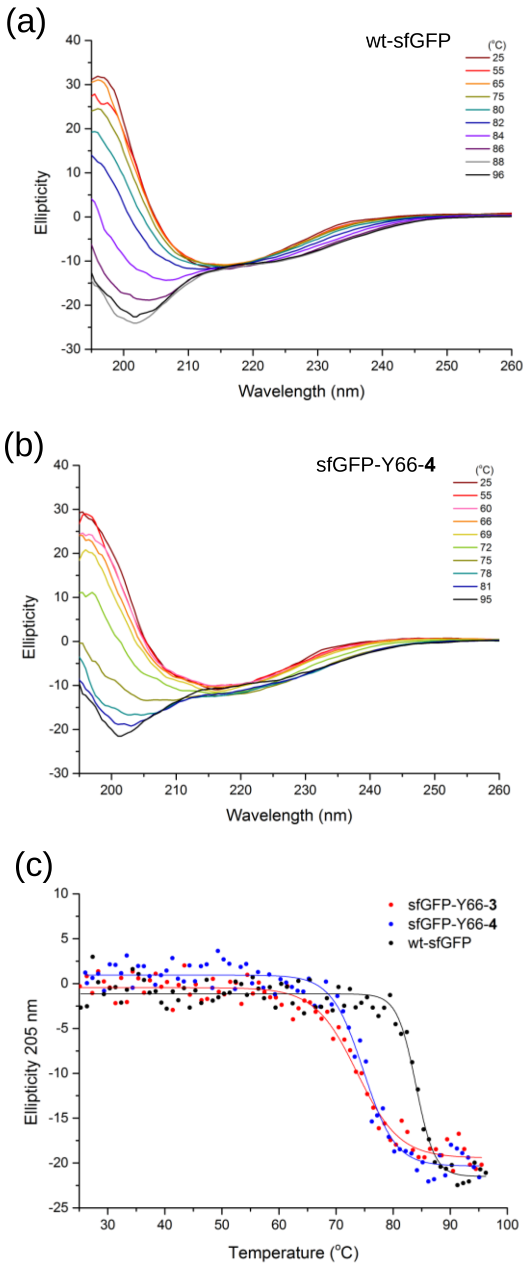

Figure S9. The CD ellipticity analysis in thermal denaturation process for sfGFP-Y66 variants.

Thermally induced conformational changes of sfGFP variants were monitored by far-UV CD analysis. The experiment was performed in 5 mM sodium phosphate buffer at pH 7.4 with 0.4 mg/mL protein concentration. The spectra of (a) sfGFP and (b) sfGFP-Y66-**4** were recorded from 25-95 °C (red to black) at a rate of 1 °C min^{-1}. A given temperature was equilibrated to keep within +/- 0.1ºC for 30 sec with the far-UV wavelength collected between 190 nm to 260 nm. (c) The melting curve of sfGFP variants were measured. The CD spectra were recorded at 205 nm between 25-95 °C with a 1 °C interval. The T_m, ΔH, ΔS, ΔG (H_2O, 298K) of sfGFP variants are listed:

Wild-type sfGFP (black): T_m, 84.4 ± 0.3 °C; ΔH, 162.5 ± 25.4 kcal mol^{-1}; ΔS, 0.45± 0.07 kcal mol^{-1} K^{-1}; ΔG, 28.3 kcal mol^{-1}.

sfGFP-Y66-**3** (red): T_m, 73.7 ± 0.4 °C; ΔH, 83.4 ± 14.0 kcal mol^{-1}; ΔS, 0.24± 0.04 kcal mol^{-1} K^{-1}; ΔG, 11.8 kcal mol^{-1}.

sfGFP-Y66-**4** (blue): T_m, 75.5 ± 0.4 °C; ΔH, 87.7 ± 12.3 kcal mol^{-1}; ΔS, 0.25± 0.04 kcal mol^{-1} K^{-1}; ΔG, 13.2 kcal mol^{-1}.

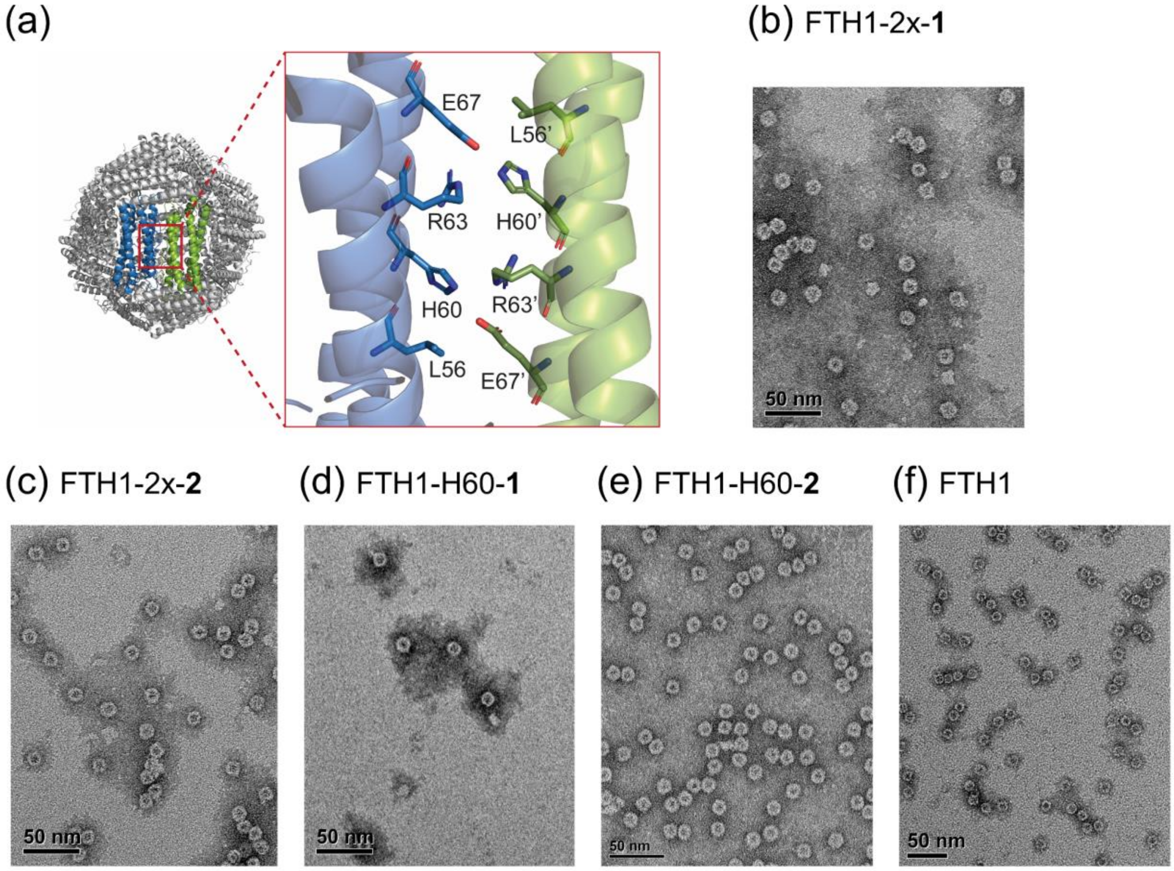

Figure S10. TEM analysis of FTH1 variants.

(a) C_2 symmetric interface of Fer. Residues L56, H60, R63, and E67 are highlighted in green sticks (PDB code: 2FHA). The Fer variants were prepared in the buffer containing 50 mM Tris-HCl (pH 8.0). TEM images of (b) FTH1-2x-**1** (50 µg/mL; particle size: 12.3 ± 1.2 nm) and (c) FTH1-2x-**2** (50 µg/mL; particle size: 12.0 ± 0.7 nm). (d) FTH1-H60-**1** (25 µg/mL; particle size: 11.6 ± 1.5 nm). (e) FTH1-H60-**2** (75 µg/mL; particle size: 12.6 ± 0.4 nm). (f) FTH1 (50 µg/mL, particle size: 11.5 ± 1.2 nm). The particle size of FTH1 variants were measured by the scale bar where n = 7. FTH1-2x-**1** and FTH1-2x-**1** indicate ferritin protein with encoded ncAAs 3-D-trifluoromethyl-phenylanine (**1**) and 3-L-trifluoromethyl-phenylanine (**2**) at H60 and E67 positions.

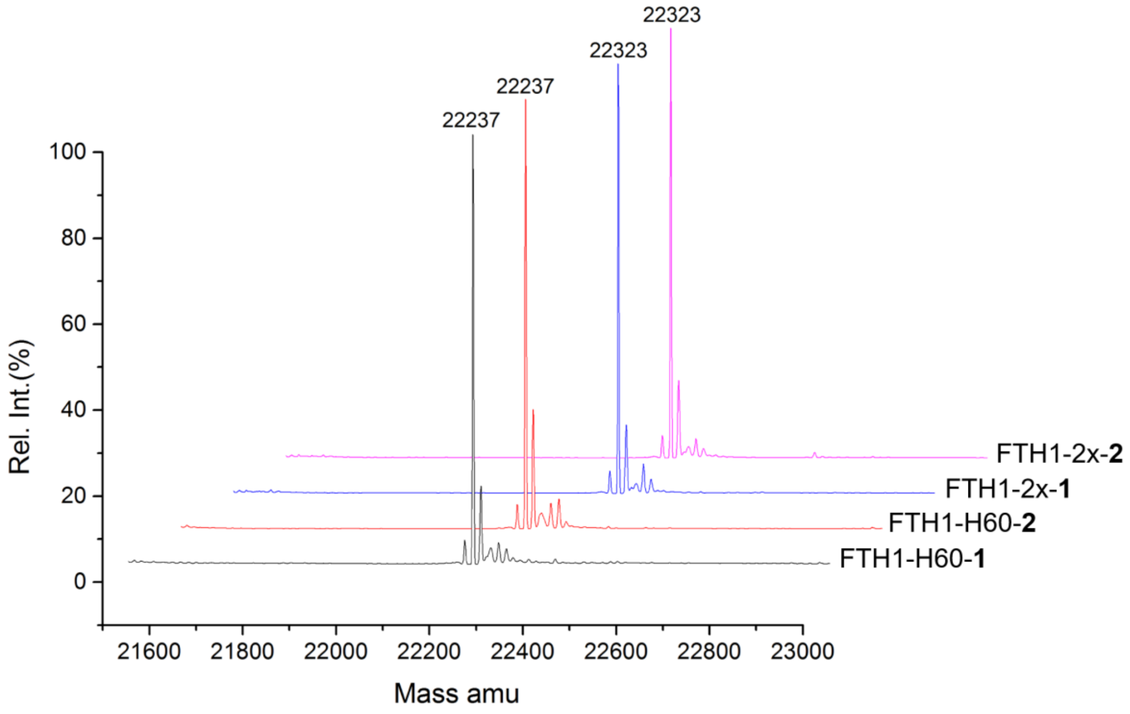

Figure S4. ESI-MS characterization of FTH1 variants.

Full-length FTH1 proteins were produced in *E. coli* BL21(DE3) cells co-expressing FTH1 containing a UAG codon at position 60 or two UAG codons at positions 60 and 63 (FTH1-2x) with the DFRS2·tRNA^{Pyl} pair with the supplement of 1 mM **1** or **2** in GMML medium at 37 °C for 12 h. The calculated masses of FTH1-H60-**1**, FTH1-H60-**2**, FFTH1-2x-**1**, and FTH1-2x-**2** are 22,237, 22,237, 22,323, and 22,323 Da (–Met). The observed masses are 22,237 Da for FTH1-H60-**1** and FTH1-H60-**2**; 22,323 Da for FTH1-2x-**1** and FTH1-2x-**2**.
